# Targeting autophagy in prostate cancer: preclinical and clinical evidence for therapeutic response

**DOI:** 10.1186/s13046-022-02293-6

**Published:** 2022-03-22

**Authors:** Milad Ashrafizadeh, Mahshid Deldar Abad Paskeh, Sepideh Mirzaei, Mohammad Hossein Gholami, Ali Zarrabi, Farid Hashemi, Kiavash Hushmandi, Mehrdad Hashemi, Noushin Nabavi, Francesco Crea, Jun Ren, Daniel J. Klionsky, Alan Prem Kumar, Yuzhuo Wang

**Affiliations:** 1 Faculty of Engineering and Natural Sciences, Sabanci University, Orta Mahalle, Üniversite Caddesi No. 27, Orhanlı, Tuzla, 34956 Istanbul, Turkey; 2 Department of Genetics, Faculty of Advanced Science and Technology, Tehran Medical Sciences, Islamic Azad University, Tehran, Iran; 3 Farhikhtegan Medical Convergence sciences Research Center, Farhikhtegan Hospital Tehran Medical sciences, Islamic Azad University, Tehran, Iran; 4 Department of Biology, Faculty of Science, Islamic Azad University, Science and Research Branch, Tehran, Iran; 5 Faculty of Veterinary Medicine, Kazerun Branch, Islamic Azad University, Kazerun, Iran; 6 Department of Biomedical Engineering, Faculty of Engineering and Natural Sciences, Istinye University, 34396 Istanbul, Turkey; 7 Department of Comparative Biosciences, Faculty of Veterinary Medicine, University of Tehran, Tehran, 1417466191 Iran; 8 Department of Food Hygiene and Quality Control, Division of Epidemiology & Zoonoses, Faculty of Veterinary Medicine University of Tehran, Tehran, Iran; 9 Department of Urological Sciences and Vancouver Prostate Centre, University of British Columbia, V6H3Z6, Vancouver, BC Canada; 10 Cancer Research Group-School of Life Health and Chemical Sciences, The Open University, Walton Hall, Milton Keynes, MK7 6AA UK; 11 Department of Laboratory Medicine and Pathology, University of Washington, Seattle, WA 98195 USA; 12 Shanghai Institute of Cardiovascular Diseases, Department of Cardiology, Zhongshan Hospital, Fudan University, Shanghai, 200032 China; 13 Life Sciences Institute & Department of Molecular, Cellular and Developmental Biology, University of Michigan, Ann Arbor, MI 48109 USA; 14 Cancer Science Institute of Singapore and Department of Pharmacology, Yong Loo Lin School of Medicine, National University of Singapore, Singapore, 117599 Singapore; 15 NUS Centre for Cancer Research (N2CR), Yong Loo Lin School of Medicine, National University of Singapore, Singapore, Singapore

**Keywords:** Anti-tumor compounds, Autophagy, Biomarker, Non-coding RNAs, Prostate cancer, Therapy response

## Abstract

Prostate cancer is a leading cause of death worldwide and new estimates revealed prostate cancer as the leading cause of death in men in 2021. Therefore, new strategies are pertinent in the treatment of this malignant disease. Macroautophagy/autophagy is a “self-degradation” mechanism capable of facilitating the turnover of long-lived and toxic macromolecules and organelles. Recently, attention has been drawn towards the role of autophagy in cancer and how its modulation provides effective cancer therapy. In the present review, we provide a mechanistic discussion of autophagy in prostate cancer. Autophagy can promote/inhibit proliferation and survival of prostate cancer cells. Besides, metastasis of prostate cancer cells is affected (via induction and inhibition) by autophagy. Autophagy can affect the response of prostate cancer cells to therapy such as chemotherapy and radiotherapy, given the close association between autophagy and apoptosis. Increasing evidence has demonstrated that upstream mediators such as AMPK, non-coding RNAs, KLF5, MTOR and others regulate autophagy in prostate cancer. Anti-tumor compounds, for instance phytochemicals, dually inhibit or induce autophagy in prostate cancer therapy. For improving prostate cancer therapy, nanotherapeutics such as chitosan nanoparticles have been developed. With respect to the context-dependent role of autophagy in prostate cancer, genetic tools such as siRNA and CRISPR-Cas9 can be utilized for targeting autophagic genes. Finally, these findings can be translated into preclinical and clinical studies to improve survival and prognosis of prostate cancer patients.

**Graphical abstract:**

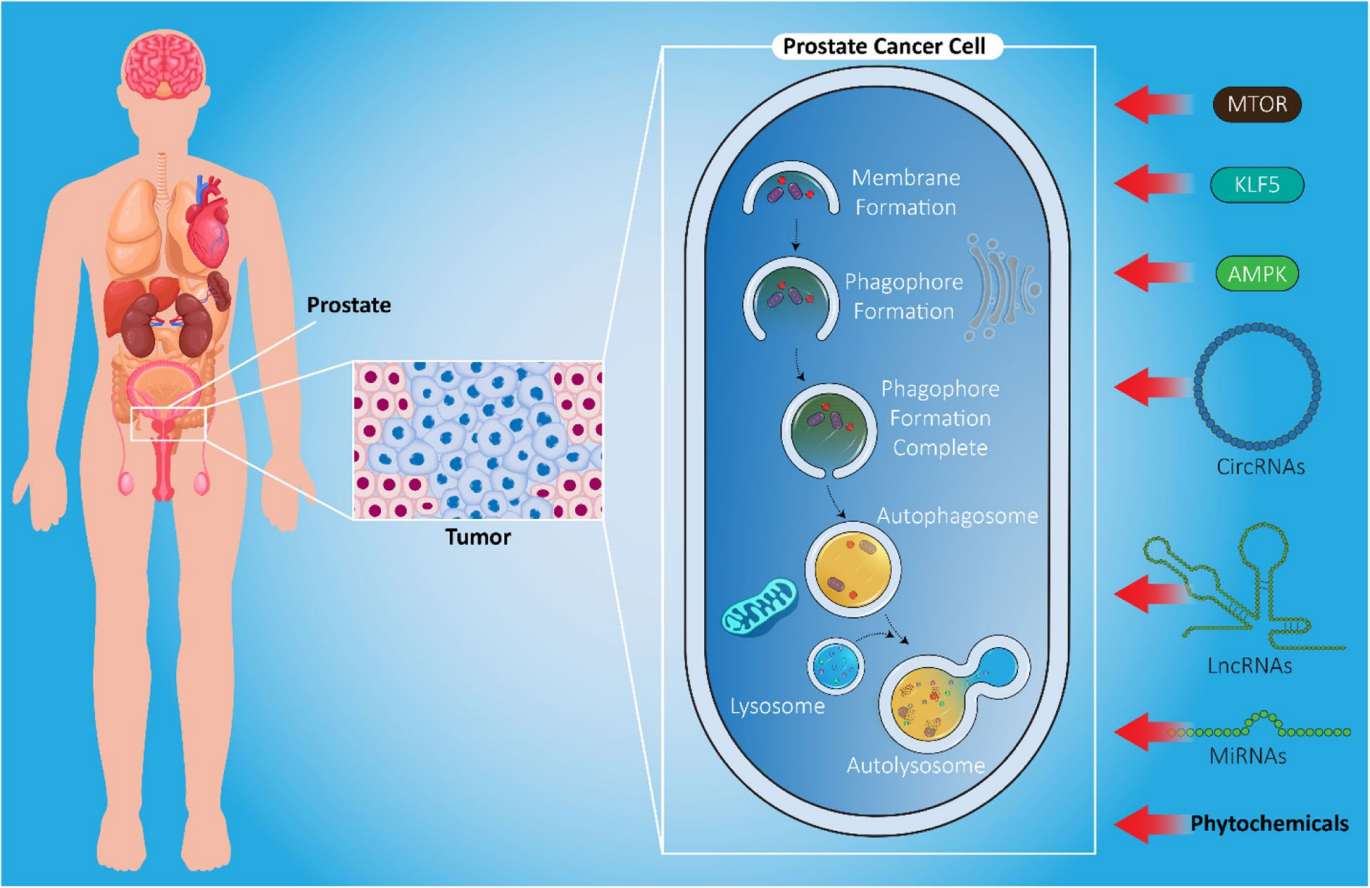

## Highlights

• Prostate cancer is among the leading causes of death in men where targeting autophagy is of importance in treatment;

• Autophagy governs proliferation and metastasis capacity of prostate cancer cells;

• Autophagy modulation is of interest in improving the therapeutic response of prostate cancer cells;

• Molecular pathways, especially involving non-coding RNAs, regulate autophagy in prostate cancer;

• Autophagy possesses both diagnostic and prognostic roles in prostate cancer, with promises for clinical application.

## Background

Prostate cancer is considered as one of the leading causes of death in men in the USA. According to the new estimates, prostate cancer is becoming the most common cause of cancer-related death, imposing a major public health concern, and novel strategies should be adopted for its prevention and cure. In 2021, it is estimated that prostate cancer will result in 31,000 deaths among 248,000 confirmed diagnoses [1, 2]. The risk of prostate cancer development is 11.6% in men [3, 4]. It should be noted that the 5-year survival rate is different among prostate cancer patients and is dependent on clinical stage, such that early stage detection results in a 5-year survival rate of more than 99%, although it is mostly incurable in advanced stages [5]. Therefore, it is of importance to develop medical tools for timely diagnosis of this malignant cancer. In most patients, prostate cancer is amenable to androgen-deprivation therapy [6–9]. However, castration-resistant prostate cancer (CRPC) develops as a result of androgen deprivation posing a major challenge in clinical treatment [10]. Different strategies have been developed for non-invasive diagnosis of prostate cancer including nanotechnology and liquid biopsies to identify circulating tumor cells, circulating nucleic acids, or exosomes, as well as other reliable biomarkers such as KLK3/prostate-specific antigen tests [11–14].

Among various factors involved in prostate cancer progression, genetic and epigenetic factors have received the major attention [15–17]. For instance, mutations in the *AR* (androgen receptor) gene leads to development of CRPC following therapy [18]. Epigenetic modifications can also lead to prostate cancer malignancy [19, 20]. Up to 20% of advanced prostate cancers have alterations and mutations in epigenetic regulators and chromatin remodelers [19, 21–23]. DNA methylation and demethylation, histone modification, REST (RE1 silencing transcription factor), and polycomb group proteins among others participate in prostate cancer development [18]. Furthermore, it has been reported that prostate cancer cells can develop therapy resistance via several molecular mechanisms including apoptosis inhibition [24–27]. Hence, it is important to reveal the molecular mechanisms in prostate cancer not only to understand progression of cancer cells, but also to determine the best response to therapy.

The aim of the current review is to provide a mechanistic viewpoint regarding the role of autophagy in prostate cancer. First, we provide a short introduction for the role of autophagy in cancer and how it affects different aspects of prostate cancer including growth, migration, and therapy response. Then, we specifically discuss the role of autophagy in prostate cancer with a focus on molecular pathways. Subsequently, we discuss anti-tumor compounds and nanotherapeutics for targeting autophagy in prostate cancer. This review should shed some light towards targeting autophagy in prostate cancer therapy.

## Autophagy machinery and its role in oncology

### Autophagy and related molecular pathways

In cells, a wide variety of physiological processes perform unique roles for maintaining homeostasis and preventing the development of pathological events [28–30]. Autophagy is a physiological mechanism first identified in the 1950s with the emergence of electron microscopy [31, 32]. This mechanism is involved in degrading cellular components by forming autophagosomes and mediating their fusion with lysosomes, resulting in cargo decomposition followed by recycling [33]. There are three primary types of autophagy, namely chaperone-mediated autophagy (CMA), microautophagy and macroautophagy. Microautophagy is the least characterized, but describes a general term for a non-selective pathway that leads to the sequestration of cytoplasmic cargos directly at the limiting membrane of the lysosome through membrane invagination [34]. CMA is a selective form of autophagy which marks individual proteins for lysosomal degradation [35]. The major form of autophagy is macroautophagy in which transient double-membrane compartments known as phagophores are produced to engulf cargoes, resulting in their subsequent containment within autophagosomes, and degradation following fusion with lysosomes. This kind of autophagy plays a significant role in maintaining cellular homeostasis [36]. Recently, much attention has been directed to deciphering the role of autophagy in cancer, and experiments show that induction and inhibition of autophagy play a significant role in cancer progression [37–43].

**Fig. 1:**
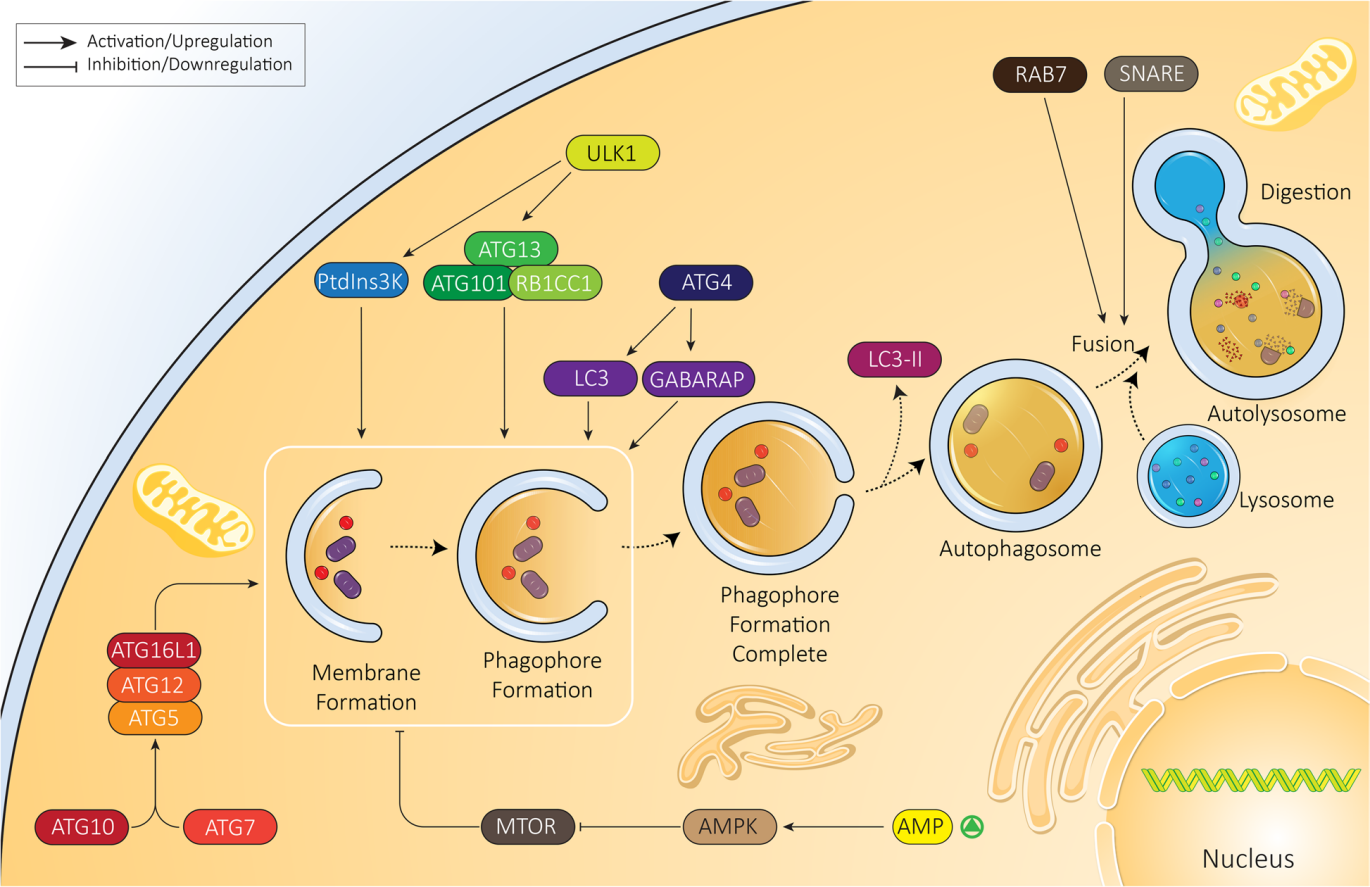
Autophagy and its regulation. The autophagy mechanism has different phases from initiation to elongation and finally, fusion with the lysosome. In each step, various molecular pathways are involved; AMPK, MTOR, and ATGs are the most well-known regulators of autophagy

Regardless of the nature of autophagy, it is essential to decipher its molecular mechanism not only for better designing future studies but also developing novel therapeutic agents [44, 45]. Overall, autophagy is divided into six steps including initiation, expansion, closure, fusion, degradation and recycling [46]. The ULK1 (unc-51 like autophagy activating kinase 1) complex containing ATG13 (autophagy related 13), ATG101 and RB1CC1 are involved in autophagy activation. The serine/threonine kinase ULK1, specifically, participates in phosphorylation of phosphatidylinositol 3-kinase (PtdIns3K) complex I components including BECN1 and PIK3C3/VPS34 and mediates phagophore production at the endoplasmic reticulum (ER) [47]. In the expansion step, the ATG12–ATG5 complex is formed through action of the ATG7 and ATG10 enzymes and is recruited to the phagophore membrane. After translation as precursor forms, MAP1LC3/LC3 (microtubule associated protein 1 light chain 3) and GABARAP proteins (representing two subfamilies referred to as Atg8-family proteins due to homology with yeast Atg8) are cleaved by ATG4. These proteolytically processed proteins are then covalently attached to phosphatidylethanolamine at the phagophore membrane in an ATG3- and ATG7-dependent process like, and involving, the generation of the ATG12–ATG5 conjugate. The next step is expansion of the phagophore for engulfing the cargo, followed by maturation of the autophagosome. At this step, LC3-II is separated from the surface of the autophagosome to complete the maturation step and allow fusion directly with a lysosome or after first fusing with an endosome [37, 48–52]. Fusion occurs with the help of molecular components that include tethering factors such as RAB7 and soluble N-ethylmaleimide-sensitive factor-activating membrane fusion protein (SNARE) proteins (Fig. 1) [53]. Finally, upon fusion with a lysosome, the contents are degraded by lysosomal enzymes, and the breakdown products are released back into the cytosol for reuse. This is the general pathway for autophagy induction and completion. Other experiments reveal the role of additional molecular pathways and signaling networks in autophagy. For instance, MTOR (mechanistic target of rapamycin kinase) is an inhibitor of autophagy. Upon MTOR activation, AMP levels are increased which lead to AMP-activated protein kinase (AMPK) activation. AMPK suppresses MTOR, inducing autophagy and increasing energy levels in the cell. In fact, complex signaling networks work with each other to ensure an appropriate function of autophagy to maintain cellular survival and homeostasis.

### Autophagy and cancer treatment

The role of autophagy in cancer is controversial and no consensus has been reached regarding the precise involvement of autophagy in cancer progression and inhibition. Autophagy can be considered as an ideal target in cancer therapeutics to ameliorate cancer mortality and morbidity. However, autophagy serves as a double-edged sword to either enhance or suppress cancer progression. A recent paper reviews the history of autophagy and cancer, noting that the role of autophagy is context dependent [37]. As autophagy plays a dual regulatory role in cancer, different experiments have investigated its participation in cancer and have provided directions for its inhibition or induction. Autophagy and apoptosis are two major branches in programmed cell death (PCD) in cancer cells. When autophagy exerts a pro-survival role, its stimulation results in a decrease in apoptosis of cancer cells, paving the way for cancer progression [54]. However, when autophagy acts as a tumor suppressor, induction of both autophagy and apoptosis can lead to cancer suppression. For instance, it was reported that MTOR inhibition by AMPK in pancreatic cancer cells stimulates autophagy in favor of potentiating apoptosis [55]. Identification of molecular pathways regulating autophagy paves the way for cancer treatment. Hypoxic conditions enhance carcinogenesis via activating pro-survival autophagy by triggering PAK1 (p21 (RAC1) activated kinase 1), which leads to subsequent phosphorylation of ATG5 [56]. These studies clearly confirm the role of autophagy in cancer proliferation [57]. Now, the question that arises is whether there is any connection between autophagy and cancer metastasis. Experimentally, the answer is positive, and autophagy can dually enhance or decrease cancer migration and invasion in various contexts [58–60]. Autophagy can significantly promote cancer metastasis via epithelial-to-mesenchymal induction. Upon autophagy inhibition, the levels of mesenchymal markers including VIM (vimentin) and CDH2/N-cadherin undergo downregulation and further confirm the role of autophagy in cancer metastasis [61].

In addition to proliferation and migration, increasing evidence demonstrates the role of autophagy in regulating immune system function [62, 63]. In ensuring tumor progression, autophagy prevents major histocompatibility complex class I (MHCI) expression and restricts anti-tumor T cell immunity against pancreatic cancer cells, resulting in immune evasion [64]. Finally, autophagy can regulate the response of cancer cells to chemotherapy; a recently conducted experiment revealed that autophagy inhibition enhances the sensitivity of gastric cancer cells to chemotherapy [65]. Overall, most available evidence agrees with the following statements:

A) Autophagy is a critical regulator of cell proliferation and metastasis;
B) autophagy can modulate the response of cancer cells to therapy;
C) anti-tumor immunity is tightly regulated by autophagy;
D) in order to develop a novel therapeutic regimen for cancer, the exact role of autophagy should be defined and, based on its role, an inhibitor or activator be recommended [66–70].

## Autophagy and prostate cancer

### Cancer proliferation

#### Autophagy increases proliferation

One of the primary effects of autophagy is promoting the proliferation and survival rate of prostate cancer cells. This is multifaceted and a result of interaction with various molecular pathways. For example, AR signaling is responsible for prostate cancer progression by inducing autophagy [71, 72]. TFEB as a transcription factor is involved in regulating lysosomal biogenesis and function. AR stimulates TFEB expression in favor of autophagy induction. Furthermore, other upstream mediators of autophagy including *ATG4B*, *ATG4D*, *ULK1* and *ULK2* are regulated by AR leading to prostate cancer progression. Further investigation reveals that AR-mediated autophagy induction is vital for proliferation and viability of prostate cancer cells and is correlated with poor prognosis [73]. Another experiment reveals the role of AR signaling in autophagy regulation in prostate cancer cells. Transcriptional regulation of GABARAPL1 (GABA type A receptor associated protein like 1), as an autophagy modulator, can be mediated via androgen, and the proliferation of prostate cancer cells is delayed by inhibiting autophagy. Androgen deprivation leads to GABARAPL1 downregulation, subsequent induction of autophagy (GABARAPL1 is autophagy repressive in this context) and increased survival and proliferation of prostate cancer cells [74]. More investigations are warranted to delineate the role of androgen deprivation therapy in prostate cancer with autophagy.

Hypoxia is a hallmark of the tumor microenvironment and can enhance cancer progression [75]. It was reported that O_2_ deprivation affects DNA replication, metastasis, and angiogenesis in favor of cancer progression [76, 77]. Hypoxic conditions lead to activation of HIF1A/HIF-1α (hypoxia inducible factor 1 subunit alpha) that subsequently enhances the proliferation and survival of prostate cancer cells [78–81]. A dual autophagy regulatory role has been found for HIF1A in cancer [82]. HIF1A exerts a tumor-promoting role in prostate cancer via affecting autophagy. At the transcriptional level, HIF1A binds to the promoter of *Atg5* to increase its expression, resulting in autophagy induction. The HIF1A-ATG5-autophagy axis is vital for tumor growth in nude mice [83]. ATG5 is a potential target for upstream mediators in autophagy induction and enhancing prostate cancer progression [84].

Different biological functions are considered for FGFs (fibroblast growth factors) including growth, apoptosis, differentiation, angiogenesis, development, and metabolic regulation [85]. FGF21 is secreted by liver and is associated with metabolic homeostasis [86]. Downregulation of FGF21 is correlated with decreased proliferation and survival suggesting an anti-tumor activity. Interestingly, through the inhibition of the PI3K-AKT-MTOR axis, FGF21 induces autophagy leading to a decrease in prostate cancer progression [87].

#### Autophagy decreases proliferation

Given the double-edged sword role of autophagy in biological events in cells, autophagy activation can reduce proliferation and survival rate of prostate cancer cells. Metabolic reprogramming in cancer cells can also influence proliferation and progression. Mitochondria play a significant role in this case. As dynamic structures, mitochondria shape, connectivity and subcellular distribution can be altered to enhance prostate cancer progression, a process modulated by DNM1L/DRP1 (dynamin 1 like) [88, 89]. A recent study has shown that DNM1L upregulation by AR signaling in prostate cancer cells affects metabolism and tumorigenesis and its downregulation results in autophagy induction and proliferation inhibition [90]. AURKA (aurora kinase A) is a serine/threonine kinase with potential roles in genetic stability by regulating spindle assembly, centrosome separation, and chromosome segregation [91, 92]. AURKA overexpression is vital for prostate cancer tumorigenesis and can function to allow tumor cells to evade therapy [93, 94]. AURKA inhibits the tumor-suppressor aspect of autophagy by suppressing AKT phosphorylation [95]. Therefore, decreasing AURKA expression can induce autophagy and suppress proliferation of prostate cancer cells. Although oxidative stress is a major pathway involved in apoptosis, it is suggested that triggering oxidative stress can also mediate autophagic cell death in prostate cancer cells [96]. The anti-tumor activity of autophagy is not only related to cell death but also other molecular pathways that are responsible for prostate cancer growth [97]. For instance, it was reported that HSD17B4 (hydroxysteroid 17-beta dehydrogenase 4) overexpression promotes proliferation and malignancy of prostate cancer cells. Acetylation of HSD17B4 promotes its degradation by CMA and leads to a subsequent decrease in prostate cancer progression [98]. In this case, autophagy does not participate in cell death, but leads to degradation of a factor that enhances prostate cancer progression.

**Fig. 2:**
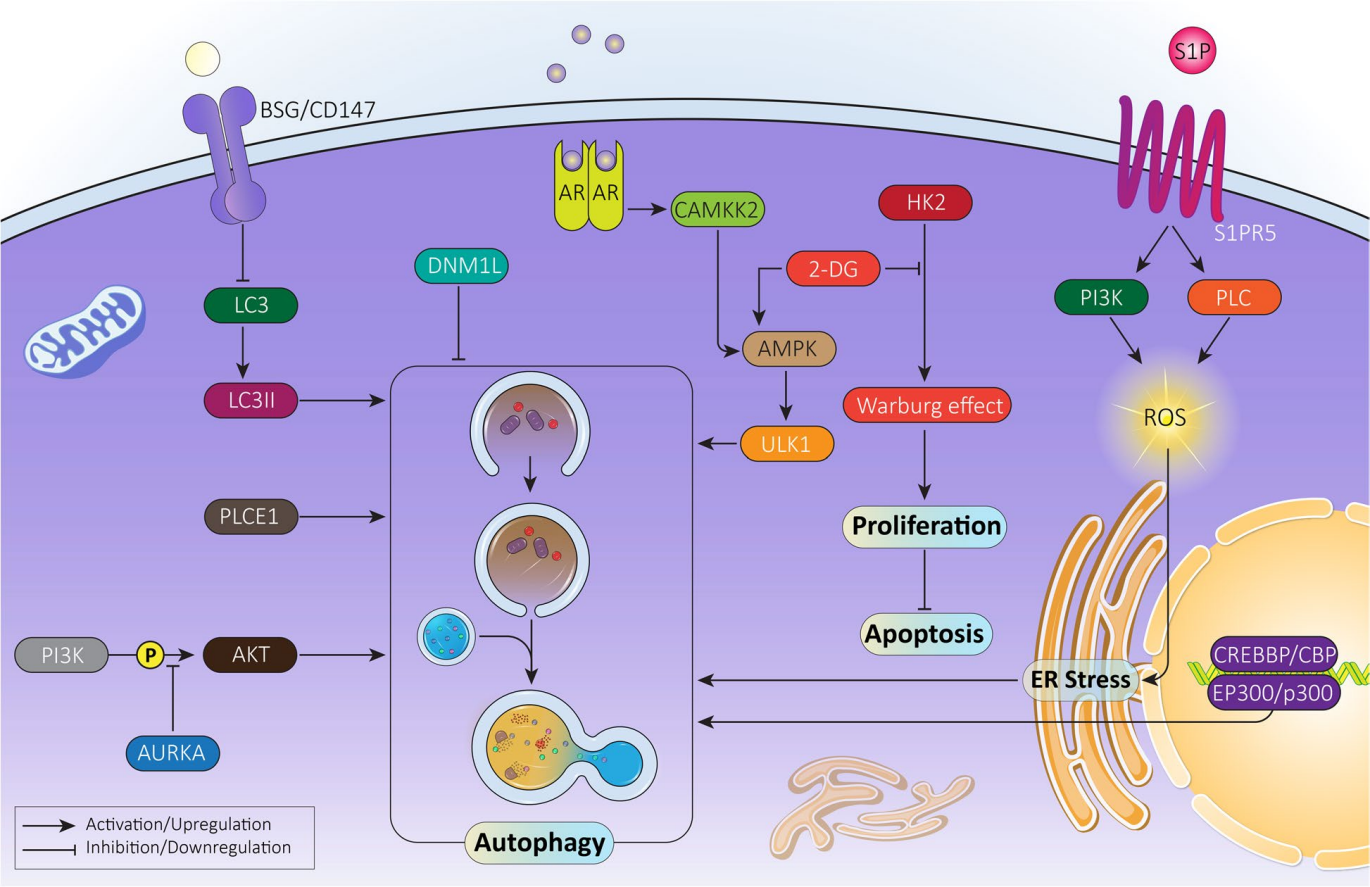
The mechanism of autophagy in prostate cancer cell proliferation and survival. Due to the dual role of autophagy, it can both promote and inhibit proliferation and viability of cancer cells. This figure provides a summary of molecular pathways involved in cancer progression regulation by autophagy

Multiple molecular pathways have been reported to regulate cell fate in cancer cells including MAPK8/JNK1/c-Jun N-terminal kinase (mitogen-activated protein kinase 8) [99, 100]. MAPK8/JNK1 activation favors cancer proliferation and enhances stem cell-like features of cancer cells [101, 102]. MAPK8/JNK1 inhibitors have been developed for cancer therapy [103–106] and specifically for prostate cancer cells, as MAPK8/JNK1 inhibits autophagy and promotes survival and proliferation of cancer cells in an androgen-independent manner. Thus, MAPK8/JNK1 inhibition results in autophagy induction and subsequent decrease in prostate cancer proliferation. The mechanism of action is suggested by an upregulation in BECN1/beclin-1 and ATG5 [107]. Overall, the following conclusions can be drawn from the studies (Fig. 2**,** Table 1):

1. Pre-clinical studies on prostate cancer reveal the dual role of autophagy in survival and proliferation;
2. autophagy not only can affect cell death, but also can influence molecular pathways involved in prostate cancer proliferation. Specifically, HSD17B4 degradation is induced by CMA;
3. autophagy can function as either a pro-survival or pro-death mechanism, and in this review, we explore targeting autophagy to suppress prostate cancer progression.

**Table 1:** Role of autophagy in proliferation and survival of prostate cancer cells

*In vitro*/*In vivo*	Cell line/Animal model	Effect on proliferation and survival	Remarks	Refs
*In vivo*	DU145 cells	Enhancement	PAK1 undergoes upregulation in prostate cancer cells and is necessary for cancer progression. Regulation of PAK1 by MTOR. Activation of MTOR promotes expression level of PAK1 and BECN1, increasing tumor growth via autophagy activation.	[108]
*In vivo* *In vivo*	PC3, LNCaP and DU145 cells Animal models	Enhancement	Inhibiting Warburg effect and simultaneous suppression of autophagy using chloroquine significantly diminishes prostate cancer progression.	[109]
*In vivo* *In vivo*	LNCaP, 22Rv1 and HEK293T cell lines Xenografts	Enhancement	CAMKK2 inhibition is associated with a decrease in prostate cancer growth via autophagy inhibition. Autophagy inhibition after CAMKK2 knockdown occurs due to AMPK-ULK1 downregulation.	[110]
*In vivo*	PC3 cells	Enhancement	ER stress induction via sphingosine-1-phosphate by enhancing ROS levels, autophagy induction and subsequent increase in prostate cancer survival.	[111]
*In vivo*	HEK293T cells	Reduction	EP300/p300-CREBBP/CBP stimulates autophagy in prostate cancer cells, providing autophagic degradation of CTNNB1/β-catenin, and a significant decrease in progression and survival of prostate cancer cells.	[112]
*In vivo*	LNCaP cells	Reduction	PLCE1/PLCe undergoes upregulation and enhances prostate cancer progression. PLCE1 enhances prostate cancer survival via AR signaling activation. PLCE1 depletion is associated with autophagy activation through the AMPK-ULK1 axis, and subsequent degradation of AR signaling to suppress prostate cancer proliferation.	[113]
*In vivo*	PC3 cells	Reduction	AR signaling inhibits autophagy in promoting prostate cancer growth. AR silencing is associated with autophagy induction and tumor growth inhibition.	[114]
*In vivo*	OC3 cells	Reduction	Overexpression of BSG/CD147 in prostate cancer cells. Silencing BSG increases GFP-LC3 puncta formation and LC3-II expression. Autophagy induction impairs proliferation and survival of prostate cancer cells.	[115]

### Cancer metastasis

Metastasis is defined as a process in which certain subpopulations of cancer cells detach and disseminate from their primary tissues or site to secondary sites [116–119]. This process is mediated via blood or lymph vasculature [120] through complex interactions with extracellular matrix (ECM), remodeling, epithelial-to-mesenchymal transition (EMT), angiogenesis, or basement membrane invasion among others to reestablish tumor colony formation in metastasis sites [121–127]. Recent experiments have also demonstrated the role of molecular pathways in migration and invasion of prostate cancer cells. For instance, one study suggests that the metastasis of prostate cancer cells into the bone is mediated via complex formations between CDK19 and CCNL1, and subsequent phosphorylation of POLR2/polymerase II at serine 2 [128]. PLEC (plectin) is another factor that induces prostate cancer metastasis to major organs of the body such as liver, lung, kidney and bone [129]. In contrast, molecular pathways that inhibit prostate cancer metastasis include the role of WFDC2 (WAP four-disulfide core domain 2), which downregulates SNAI/snail expression and inhibits EGFR (epidermal growth factor receptor) [130]. These studies have highlighted the fact that metastasis is a critical challenge in the treatment of prostate cancer, and a variety of complex molecular pathways and mechanisms are involved in this process. In this section, the role of autophagy in prostate cancer metastasis is discussed.

Metastatic prostate cancer cells display induction in autophagy. Thus, there is a vital role for autophagy in metastasis and migration. Tumor microenvironment (TME) contains a variety of non-malignant cells such as inflammatory cells, cancer-associated fibroblasts and vascular cells [131]. It has been reported that among the TME constituents, endothelial cells play a significant role in prostate cancer metastasis. After androgen-deprivation therapy (ADT), apoptosis occurs in prostatic microvasculature, although the endothelial cells undergo immediate regeneration [132]. An increase in microvascular infiltration is associated with prostate cancer metastasis through AR signaling [133, 134]. In vivo and in vitro experiments showed that autophagy is associated with prostate cancer metastasis. For this to occur, endothelial cells inhibit AR signaling to provide autophagy induction. Subsequently, autophagy activation leads to a rise in migration and invasion of prostate cancer cells through focal adhesion protein disassembly [135].

With respect to the dual role of autophagy, metastasis of prostate cancer cells can also be suppressed. HADC6 (histone deacetylase 6) has a close relationship with autophagy in cells. The autophagy maturation by HDAC6 induces cancer suppression [136]. Furthermore, HDAC6 can inhibit autophagy via TUBA/α-tubulin deacetylation [136]. In prostate cancer cells, microtubule acetylation results in induction of autophagosome formation and autophagy flux to suppress migration and invasion. However, SQSTM1/p62 acts as a tumor-promoting factor, inducing HDAC6 expression to suppress microtubule acetylation-mediated autophagy, leading to prostate cancer metastasis [137]. This experiment reveals that the role of autophagy in suppressing prostate cancer metastasis and triggering autophagy flux is of importance in this case.

**Fig. 3:**
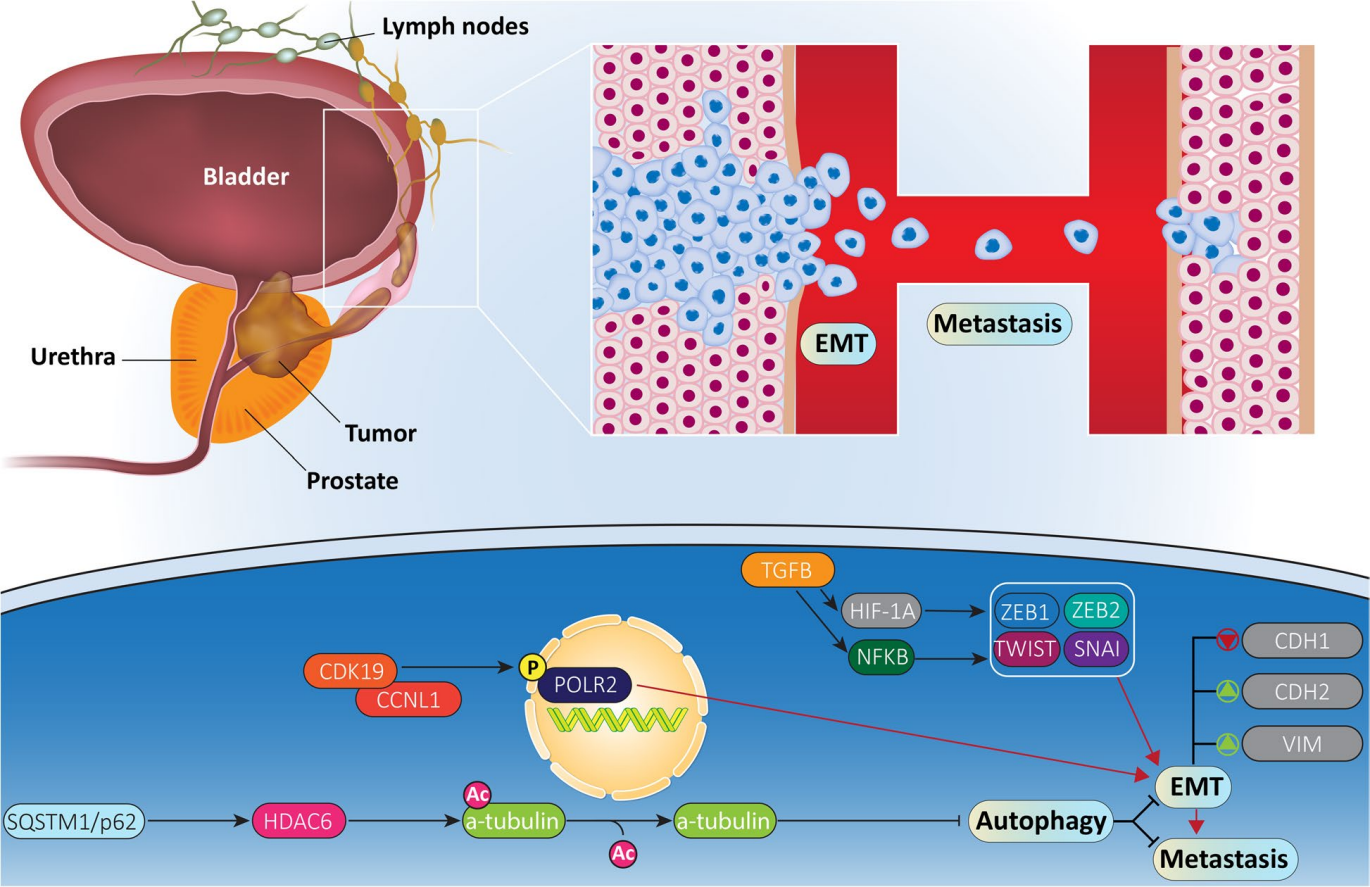
The mechanism of autophagy in prostate cancer metastasis. In addition to proliferation, migration of prostate cancer cells is regulated by autophagy. As shown, upstream mediators can induce EMT-mediated metastasis of prostate cancer cells, and autophagy is capable of suppressing EMT and invasion

As more reports have surfaced, more molecular mechanisms involved in prostate cancer metastasis are revealed. One of the most important mechanisms in cancer metastasis is EMT occurring due to decrease in CDH1/E-cadherin levels and increase in CDH2/N-cadherin and VIM levels [138–140]. Upon EMT induction, cancer cells acquire a mesenchymal phenotype via losing their cellular polarity and adhesion to basement membrane [138, 139, 141]. TGFB1/TGF-β (transforming growth factor beta 1) is considered as main inducer of EMT via upregulation of NFKB/NF-κB (nuclear factor kappa B) and HIF1A [142–144]. Furthermore, TWIST, ZEB proteins and SNAI are among other factors involved in EMT induction [145]. Similarly, in prostate cancer cells, different molecular pathways such as STAT3 (signal transducer and activator of transcription 3) and microRNAs (miRNAs) participate in EMT regulation [146, 147]. Conversely, autophagy can regulate EMT in cancer cells [148]. In prostate cancer cells, SGK1 (serum/glucocorticoid regulated kinase 1) as a tumor-promoting factor, increases migratory ability in vivo and in vitro via EMT induction. Silencing SGK1 impairs prostate cancer metastasis via autophagy induction and subsequent suppression of EMT. It was indicated that autophagy inhibition of EMT in prostate cancer occurs via SNAI downregulation [149]. These studies highlight the fact that autophagy is in close association with prostate cancer metastasis and vital mechanisms such as EMT can be affected in this way. Conversely, upstream mediators target autophagy in regulating metastasis of prostate cancer cells (Fig. 3).

### Therapeutic resistance

In the field of cancer therapy, especially prostate cancer treatment, drug resistance remains an increasing challenge [150, 151]. A variety of molecular pathways and mechanisms can result in drug resistance development such as activation of tumor-promoting factors and inhibition of tumor-suppressor factors [152–154]. In respect to emergence of genetic tools such as small interfering RNA (siRNA), short-hairpin RNA (shRNA) and the CRISPR-Cas9 system for gene regulation, molecular mechanisms involved in drug resistance can be targeted in providing cancer sensitization to chemotherapy [155–158]. Autophagy, owing to its dual role, can either suppress or induce chemoresistance [159]. In some cases, inhibition of autophagy by *MIR375* and WWOX (WW domain containing oxidoreductase) can promote chemosensitivity of cancer cells [160, 161]. Noteworthy, TME provides an environment favoring autophagy induction and chemoresistance [162]. Conversely, there is also evidence demonstrating that autophagy induction participates in elevated efficacy of chemotherapy in cancer elimination [163, 164]. In addition to chemotherapy, autophagy affects the response of cancer cells to radiotherapy. In this case, autophagy can also induce or inhibit radio-resistance [165–167]. Due to the aggressive behavior of prostate cancer cells, these malignant cells obtain resistance to radio- and chemotherapy [168, 169]. In this section, a mechanistic discussion of autophagy in the therapy response of prostate cancer cells is provided to direct the next experiments towards modulating this important molecular pathway in effective treatment of prostate cancer.

#### Autophagy as a pro-survival mechanism

Analysis of NPRL2/nitrogen permease regulator-like 2 (NPR2 like, GATOR1 complex subunit) demonstrates modulatory impacts on autophagy. NPRL2 can induce autophagy via MTOR signaling inhibition [170–172], and autophagy activation can promote resistance of cancer cells to everolimus [173, 174]. In CRPC cells, NPRL2 shows an increase in expression and is associated with proliferation and drug resistance features. Upon upregulation, NPRL2 suppresses MTOR signaling to stimulate autophagy, resulting in everolimus resistance via reducing apoptotic cell death [175]. This observation demonstrates that apoptosis and autophagy as two major arms of PCD are in close relationship and autophagy, as a tumor-promoting factor, can alleviate apoptosis induction in prostate cancer cells.

One of the important findings is the activation of autophagy by chemotherapeutic agents in mediating resistance. In fact, this unwanted response of chemotherapeutic agents negatively affects their anti-tumor activity. It has been reported that autophagy induction by NPRL2 leads to docetaxel resistance [176]. Exposing CRPC cells to docetaxel is associated with autophagy activation via BECN1 upregulation. Autophagy inhibition via MTOR signaling induction significantly enhances therapeutic efficacy of docetaxel in prostate cancer treatment [177]. TGFB is an upstream mediator of autophagy, and TGFB1 fucosylation induces autophagy [178]. It seems that TGFB and autophagy interaction is of importance for docetaxel resistance of prostate cancer cells. In this way, mesenchymal stem cells (MSCs) along with docetaxel induce autophagy to diminish proliferation inhibition and apoptosis induction in prostate cancer cells. It was reported that autophagy inhibition suppresses docetaxel resistance. In autophagy induction, MSCs secrete TGFB1 and preventing TGFB1 secretion inhibits autophagy, leading to increased docetaxel sensitivity of prostate cancer cells [179]. KLF5 is a component of another molecular pathway capable of regulating autophagy and docetaxel sensitivity of prostate cancer cells. As a transcription factor, KLF5 is widely distributed in various organs such as liver, kidney, prostate and colon with vital roles in regulating proliferation and tumorigenesis [180]. The *KLF5* gene is located on chromosome 13q.21 and genomic hybridization analysis has demonstrated its deletion in 39% of prostate cancer cases [181]. There is a negative relationship between autophagy and KLF5 in cancer, so that KLF5 provides poor prognosis and autophagy inhibition [182]. However, a recent study depicted a tumor-suppressor role for KLF5 in prostate cancer. KLF5 downregulation is associated with unfavorable prognosis and decreased sensitivity of prostate cancer cells to docetaxel. Furthermore, In vivo and in vitro studies have demonstrated KLF5 downregulation upon docetaxel exposure. In fact, docetaxel reduces KLF5 expression to prevent cell death induction in prostate cancer cells. Examination of molecular pathways demonstrates that KLF5 inhibits BECN1 and HDAC3 (histone deacetylase 3) cooperation to suppress autophagy and promote docetaxel sensitivity in the context of prostate cancer. Besides, docetaxel exposure downregulates KLF5 expression via an AMPK-MTOR-RPS6KB/p70S6K axis to increase BECN1 expression, leading to autophagy induction and chemoresistance features of prostate cancer cells [183]. AMPK upregulation stimulates protective autophagy to provide survival and viability of prostate cancer cells against AD therapy [184].

Cisplatin (CP) is another well-known chemotherapeutic agent applied in prostate cancer therapy. Increasing cell stiffness, decreasing migration, and apoptosis induction result from treatment [185, 186]. However, activation in certain cell signaling cascades such as activation of the WNT-CTNNB1/beta-catenin and AKT pathways can result in CP resistance of prostate cancer cells [187, 188]. AMBRA1 (autophagy and beclin 1 regulator 1) can induce autophagy via regulating BECN1 and BCL2 [189, 190]. In prostate cancer cells. AMBRA1 undergoes overexpression and protects prostate cancer cells against CP-mediated apoptosis, and CASP3 (caspase 3)-mediated PARP (poly(ADP-ribose) polymerase) cleavage. By increasing autophagy, AMBRA1 prevents apoptosis in prostate cancer cells and enhances their colony formation, leading to CP resistance [191].

ASAH1/acid ceramidase (N-acylsphingosine amidohydrolase 1) is an enzyme localized in lysosomes capable of metabolizing ceramide, an intermediate in sphingolipid synthesis. Ceramide is involved in the regulation of molecular pathways such as apoptosis, cell cycle arrest and inflammation. It has been reported that changes in ceramide metabolism can provide drug resistance feature of cancer cells [192, 193]. Prostate cancer cells demonstrate overexpression of ASAH1 converting ceramide to sphingosine and sphingosine-1-phosphate, inhibiting apoptosis [194]. ASAH1 upregulation also enhances lysosomal density and results in autophagy induction. Autophagy inhibition can promote a therapeutic response of prostate cancer cells to C (6) ceramide. It is possible that autophagy can provide resistance of prostate cancer cells to SRC-family kinase inhibitors. SRC is a large family of nonreceptor tyrosine kinases, and its overexpression occurs in various cancers, particularly prostate cancer. Activation of SRC signaling is associated with recurrence of prostate cancer [195]. Besides, SRC mediates metastasis of prostate cancer cells in hypoxic condition [196]. Therefore, SRC inhibitors have been developed for prostate cancer therapy, but drug resistance has restricted their potential. It seems that autophagy is involved in resistance of prostate cancer cells to SRC inhibitors. SRC inhibition does not affect autophagy, showing that autophagy induction is independent of SRC upregulation. Using chloroquine as an autophagy inhibitor enhances sensitivity of prostate cancer cells to SRC inhibitors [197]. In fact, the anti-tumor activity of some compounds is boosted when protective autophagy is inhibited [198].

STAT3 mainly possesses a tumor-promoting role in cancer, and its downregulation is beneficial for cancer treatment [140, 199–202]. Conversely, STAT3 can function as an upstream mediator of autophagy in cancer [203–205]. Following docetaxel chemotherapy, autophagy activation occurs via upregulation of BECN1 and induction of the BECN1-PIK3C3/VPS34-ATG14 complex. It seems that STAT3 is a negative regulator of autophagy in prostate cancer cells upon docetaxel chemotherapy. Autophagy induction is vital for resistance to docetaxel chemotherapy. Enhancing the expression level of STAT3 suppresses autophagy that may promote docetaxel sensitivity of prostate cancer cells [206]. Similar to STAT3, HMGB1 (high mobility group box 1) can regulate a wide variety of biological mechanisms such as differentiation, autophagy and migration [207, 208]. HMGB1 overexpression promotes prostate cancer invasion via EMT induction [209]. Besides, HMGB1 can trigger chemoresistance feature of prostate cancer cells via activating downstream targets such as MYC/c-Myc signaling [209]. The association between HMGB1 and autophagy is in favor of triggering gemcitabine resistance of prostate cancer cells. In this way, exposing prostate cancer cells to gemcitabine significantly enhances the expression level of HMGB1. Downregulating HMGB1 via shRNA diminishes autophagy. Therefore, HMGB1 induces autophagy to protect prostate cancer cells against gemcitabine-mediated cell death [210]. In hormone-refractory prostate cancer cells, autophagy induction is of importance for cancer progression [211]. Although androgen deprivation is a promising therapy for prostate cancer, providing such conditions for prostate cancer cells induces protective autophagy to prevent apoptosis [212].

#### Autophagy as a pro-death mechanism

When autophagy possesses an anti-tumor role, its activation significantly enhances sensitivity of cancer cells to therapy. In the previous section, it was discussed that autophagy induction promotes docetaxel resistance in prostate cancer cells. Autophagy stimulation can also be helpful in docetaxel sensitivity via promoting apoptosis. TPD52/PrLZ/prostate leucine zipper (tumor protein D52) localized on chromosome 8q21.1 has specific expression in prostate tissues and it demonstrates upregulation in advanced prostate cancer [213]. In respect to the tumor-promoting role of TPD52, its activation remarkably promotes progression of prostate cancer cells [214]. A recent experiment has shown involvement of TPD52 in docetaxel resistance of prostate cancer cells via inhibiting autophagy and subsequent docetaxel-mediated apoptosis. Exposing prostate cancer cells to docetaxel inhibits TPD52 and STK11/LKB1 (serine/threonine kinase 11) interaction to promote STK11 expression. Then, STK11 as an upstream mediator of AMPK signaling, induces autophagy to potentiate apoptosis in cancer cells. However, TPD52 upregulation in drug resistant-prostate cancer cells leads to STK11 and AMPK downregulation and subsequent inhibition of autophagy [215].

**Fig. 4:**
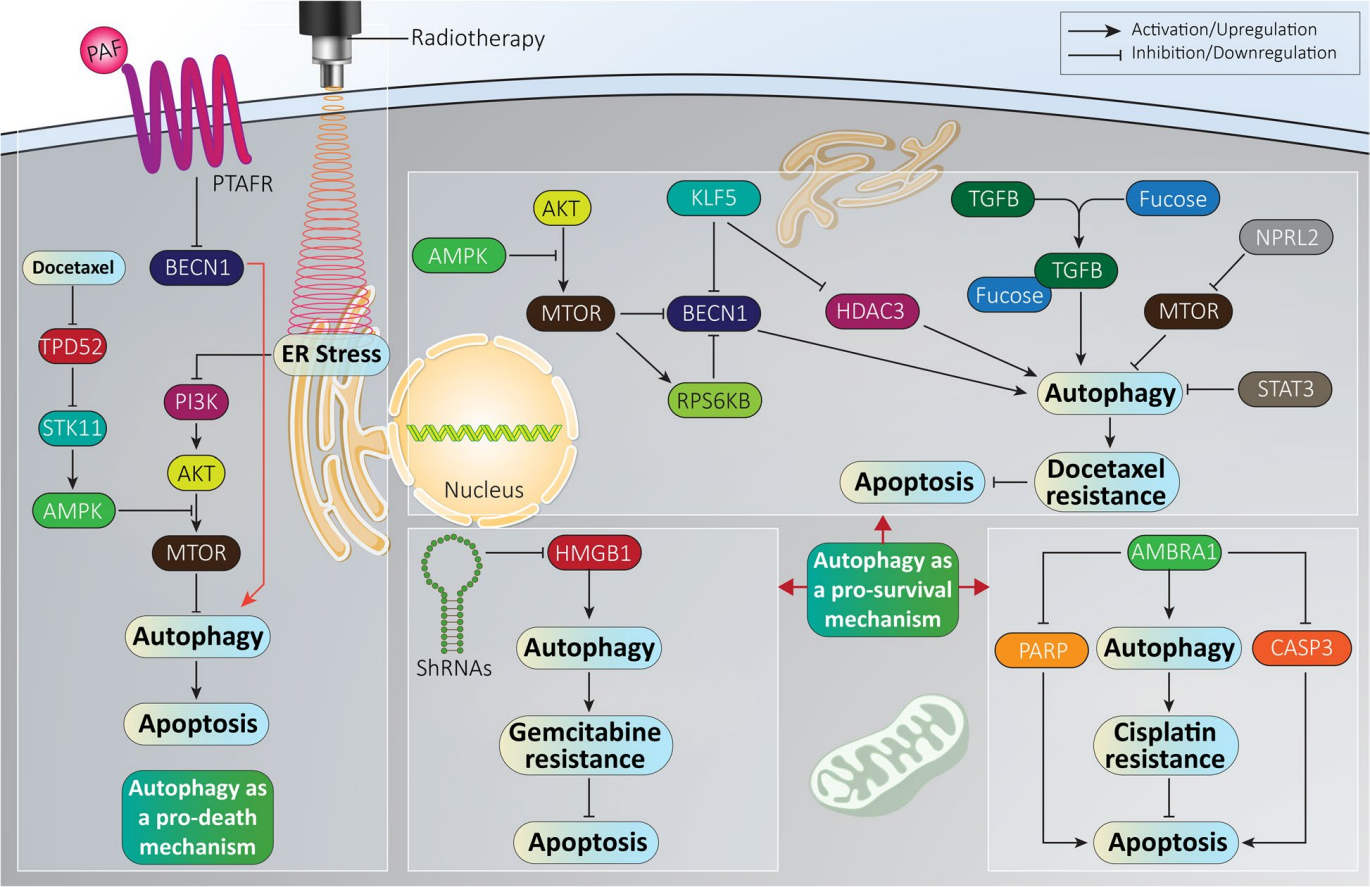
Autophagy regulates the response of prostate cancer cells to therapy. An increasing challenge in prostate cancer therapy is therapy resistance. On the one hand, autophagy activation as a tumor-promoting factor, can inhibit apoptosis and mediate chemoresistance. On the other hand, tumor-suppressor autophagy can sensitize prostate cancer cells to chemotherapy via triggering apoptosis

PTAFR (platelet activating factor receptor) belongs to the G-protein-coupled receptor (GPCR) family with vital roles in cancer progression via regulating molecular pathways such as STAT3 and PI3K-AKT signaling networks [216]. PTAFR plays a significant role in mediating the radio-resistance feature of prostate cancer cells. In this way, PTAFR destabilizes BECN1 to suppress autophagy. Interfering with the PTAFR-BECN1 complex is of importance in enhancing the radio-sensitivity of prostate cancer cells (Fig. 4**,** Table 2) [225].

**Table 2:** Autophagy as a regulator of therapy response of prostate cancer cells

Autophagy role	Therapy response	Remarks	Refs
Pro-survival	Docetaxel resistance	FOXM1 induces chemoresistance of prostate cancer cells in vivo and in vitro*.* Increased autophagy flux and formation of autophagosomes. Targeting the AMPK-MTOR axis in favor of autophagy induction. FOXM1 enhances docetaxel sensitivity of prostate cancer cells.	[217]
Pro-survival	AKT inhibitor resistance	AKT inhibitor AZD5363 induces both cell cycle arrest and autophagy, but no significant apoptosis induction observed. Autophagy inhibition using lysosomotropic agents leads to higher potential of AKT inhibitors in prostate cancer therapy.	[218]
Pro-survival	Cisplatin resistance	Silencing CFTR inhibits autophagy to promote cisplatin sensitivity of prostate cancer cells. Stimulation of AKT-MTOR signaling occurs after CFTR downregulation.	[219]
Pro-death	Radiotherapy	Decreased colony formation using a combination of gamma irradiation and photodynamic therapy. Decreasing cell viability. Inducing necrosis and autophagy, but not apoptosis.	[220]
Pro-death	Radiotherapy	MTOR inhibition leads to autophagy induction and enhanced sensitivity to radiotherapy. Apoptosis blockade or caspase inhibition potentiates autophagy induction.	[221]
Pro-death	Radiotherapy	FBP1 downregulation results in autophagy stimulation via the AMPK-MTOR axis. Removing protective autophagy and enhancing radio-sensitivity.	[222]
Pro-death	Paclitaxel sensitivity	Autophagy induction upon exposing prostate cancer cells to ultrasound (sonodynamic therapy). Inducing ER stress. Inhibiting the PI3K-AKT-MTOR axis via ER stress and subsequent autophagy stimulation.	[223]
Pro-death	Immunotherapy	ESK981 diminished viability of cancer cells. Inducing cell cycle arrest at G2/M phase. Inhibition of autophagy flux by ESK981.	[224]

### Molecular pathways regulating autophagy in prostate cancer

#### MicroRNAs

A variety of molecular pathways can function as upstream mediators of autophagy and miRNAs are among them. MiRNAs are a conserved class of non-coding RNAs (ncRNAs) 18–22 nucleotides in length. Although miRNAs do not encode proteins, they participate in the regulation of many protein-coding genes (up to 60%). Furthermore, miRNAs downregulate expression of target gene via targeting messenger RNA (mRNA) [226–228]. MiRNAs are supposed to be expressed in all tissues [145, 229–231]. For affecting expression of a target gene, first miRNAs cooperate with specific proteins, known as AGO (argonaute) proteins, and then they are embedded in an RNA-induced silencing complex (RISC) to bind to the 3^/^−untranslated region (3′-UTR) of mRNA, suppressing expression via inhibiting translation, inducing mRNA cleavage or enhancing mRNA stability [232]. Increasing evidence demonstrates a role for miRNAs in the regulation of various molecular pathways and mechanisms in physiological and pathological conditions [233, 234]. MiRNAs are potential regulators of autophagy in cancers, affecting proliferation, metastasis, and therapy response [235, 236]. In this section, a mechanistic discussion of autophagy regulation by miRNAs in prostate cancer is provided to shed more light on their interaction and contribution to cancer progression in malignant cells.

Molecular pathways regulating autophagy are affected by miRNAs in prostate cancer. It is worth mentioning that miRNAs with a tumor-suppressing role undergo downregulation during cancer progression. It has been reported *MIR26B* is present at a low level in prostate cancer cells. This miRNA downregulates the expression level of *ULK2* by binding to its 3′-UTR to suppress autophagy, leading to prostate cancer progression impairment [237]. In contrast, when autophagy possesses a tumor-suppressing role, its inhibition occurs by tumor-promoting miRNAs. *MIR146B* is considered as a tumor-suppressing factor, which inhibits proliferation and invasion of prostate cancer cells, and induces apoptosis [238]. However, a recent report has revealed the tumor-promoting role of *MIR146B* and its upregulation in prostate cancer, demonstrating a double-edged sword role of this miRNA [239]. PI3K affects autophagy via regulating MTOR. Of note, PTEN (phosphatase and tensin homolog) is an upstream and negative regulator of PI3K-AKT signaling [240, 241]. PTEN downregulates PI3K-AKT to prevent proliferation and invasion of cancer cells and promote their therapy response [242–247]. *MIR146B* downregulates expression of PTEN to induce AKT-MTOR signaling. Then, autophagy inhibition occurs to ensure progression of prostate cancer [239]. RELN (reelin) is another regulator of PI3K-AKT signaling, and in contrast to PTEN, RELN induces this pathway [248]. By downregulating AR expression, *MIR381* suppresses proliferation and progression of prostate cancer cells [249]. It is worth noting that there is a close association among *MIR381*, RELN and the PI3K-AKT-MTOR axis in prostate cancer cells. In respect to the tumor-suppressing role of *MIR381*, this factor downregulates RELN to inhibit PI3K-AKT-MTOR signaling, leading to autophagy and apoptosis induction [250].

*MIR493* is another miRNA with its role in prostate cancer not fully elucidated. Although one independent study has demonstrated the anti-tumor activity of *MIR493* in prostate cancer via upregulation of N^6^-methyladenosine levels [251], another study revealed that *MIR493* stimulates AKT signaling to enhance prostate cancer proliferation [252]. Therefore, more studies are required to reveal role of this miRNA in prostate cancer. However, *MIR493* also exerts a tumor-suppressing role via autophagy regulation in prostate cancer. *MIR493* upregulates PHLPP2 (PH domain and leucine rich repeat protein phosphatase 2) to promote expression of BECN1 and ATG7, resulting in autophagy and reduced ability of prostate cancer cells in colony formation [253]. Studies demonstrate that upstream mediators of autophagy such as MTOR and certain ATGs are tightly regulated by miRNAs in prostate cancer. For instance, *MIR139* induces MTOR signaling, while downregulating BECN1 to inhibit autophagy flux, resulting in subsequent apoptosis induction in prostate cancer cells [254].

More importantly, miRNAs targeting autophagy can regulate the response of prostate cancer cells to therapy. In previous sections, it was shown how autophagy can affect the therapy response of cancer cells. Radio-resistance is an increasing in treatment for prostate cancer. Activation of DNA repair mechanisms, and downregulating tumor-suppressing miRNAs such as *MIR501-3p* participate in the radio-resistance feature of prostate cancer cells [255, 256]. Identification of miRNAs regulating autophagy in prostate cancer and its association with radiotherapy response are of importance in developing novel therapeutics to overcome this issue. Exposing prostate cancer cells (DU145 and LNCaP cells) to irradiation induces protective autophagy via TP53INP1 (tumor protein p53 inducible nuclear protein 1). *MIR205* diminishes the radio-resistance of prostate cancer cells via downregulating TP53INP1, inhibiting autophagy and causing a subsequent impairment in viability and survival [257]. *MIR32* is another factor that upregulates BECN1 and LC3-II, while downregulating MTOR to induce autophagy and provide radio-resistance in prostate cancer cells [258].

One of the hallmarks of cancer is hypoxia in the tumor microenvironment. The hypoxic condition is induced due to low vasculature and the presence of cancer cells with a rapid growth rate. Therefore, molecular pathways such as those involving HIF1α and angiogenesis are activated to ensure cancer progression and growth. In prostate cancer cells, SP1 (Sp1 transcription factor) induces hypoxia-mediated autophagy to promote cancer progression. It is noteworthy that SP1 regulates PKM2 (pyruvate kinase M1/2) in this manner. As a tumor-suppressing factor, *MIR361-5p* inhibits SP1 and its downstream target PKM2 to impair autophagy and cancer progression of prostate cancer cells [259]. It seems that the expression level of miRNAs with anti-tumor activity decreases in hypoxic conditions; in these conditions, *MIR30A* and *MIR205* undergo downregulation to promote prostate cancer progression. Then, autophagy activation occurs to protect these malignant cells against irradiation. However, enhancing levels of *MIR30A* and *MIR205* downregulate the level of TP53INP1 via binding to its 3′-UTR to suppress autophagy, resulting in an increased radio-sensitivity of prostate cancer cells [260].

*MIR212* can dually induce or inhibit prostate cancer progression. This dual role of miRNAs has made it rather complicated to target miRNAs in prostate cancer therapy. For instance, *MIR212* downregulates MAPK expression to suppress prostate cancer proliferation [261], and at the same time, this miRNA can induce NFKB signaling in prostate cancer development [262]. On a related note, there is a connection between SIRT1 (sirtuin 1) and autophagy [263], and SIRT1 can function as an autophagy inducer [264]. A significant decrease occurs in levels of circulatory *MIR212* in the serum of prostate cancer patients. Investigation of molecular pathways demonstrates downregulation of SIRT1 by *MIR212* and subsequent inhibition of autophagy that are of importance for suppressing prostate cancer progression [265].

ERN1/IRE1 (endoplasmic reticulum to nucleus signaling 1) is a marker of ER stress and its upregulation can also be associated with autophagy [266, 267]. During starvation, ER stress occurs and upon ERN1 upregulation autophagy is activated to restore homeostasis. In prostate cancer cells, *MIR200C-3p* induces autophagy via ERN1 upregulation [268]. With respect to the tumor-promoting role of autophagy in prostate cancer, the interaction among miRNAs and ER stress-related markers in regulating autophagy should be highlighted. Considering everything together, the following bullet points can be concluded:

1. Both tumor-suppressing and tumor-promoting miRNAs can regulate autophagy in affecting prostate cancer progression;
2. upstream mediators of autophagy such as SIRT1, BECN1, ATGs and LC3-II are regulated by miRNAs in prostate cancer;
3. there is a close relationship between ER stress and autophagy markers that can be regulated by miRNAs;
4. The tumor microenvironment of prostate cancer cells affects the expression level of miRNAs in favor of regulating autophagy for progression and survival;
5. miRNA replacement therapy and, small interfering RNAs (siRNAs) can be utilized for affecting the expression level of miRNAs and their downstream targets, respectively to provide conditions for autophagy regulation and suppressing progression of prostate cancer cells.

#### LncRNAs

As transcripts with more than 200 nucleotides in length, lncRNAs are another key member of ncRNAs with vital physiological roles in development and differentiation [269–272]. Despite their expression in various major organs of the body such as heart, kidney, and liver, it seems that expression of lncRNAs is tissue specific and is maintained at a low level of expression [273–276]. LncRNAs do not encode proteins, but they have potential and important functions like protein-coding genes [277]. Genomic imprinting, apoptosis, differentiation, splicing, cell cycle regulation and epigenetic regulation are among the vital roles of lncRNAs in cells [278–280]. Dysregulation of lncRNAs is a common phenomenon in cancer due to their role in regulating important biological processes [281]. In prostate cancer, lncRNAs can regulate lymph node metastasis, proliferation, and therapy response via affecting different molecular pathways, especially miRNAs [282–284]. In this section, the role of lncRNAs in regulating autophagy and affecting prostate cancer progression is discussed.

*SNHG1* (small nucleolar RNA host gene 1) is a new emerging lncRNA located on chromosome 11 that plays a key role in cancer. The role of *SNHG1* in prostate cancer is suggested to be tumor-promoting, so that *SNHG1* can increase metastasis via EMT induction [285]. In addition, *SNHG1* can enhance proliferation and survival of prostate cancer cells via AKT2 upregulation [286]. About promoting prostate cancer progression, *SNHG1* can affect autophagy. In this way, *SNHG1* induces PI3K-AKT signaling to induce MTOR expression, resulting in autophagy inhibition, paving the way for survival and proliferation of prostate cancer cells. It is likely that *SNHG1* indirectly affects PI3K-AKT signaling, so that first, *SNHG1* promotes the expression level of EZH2 (enhancer of zeste 2 polycomb repressive complex 2 subunit) to affect PI3K-AKT signaling. Silencing *SNHG1* or EZH2 is associated with decreased viability of prostate cancer cells. Further investigation reveals regulation of WNT-CTNNB1β-catenin signaling by the *SNHG1*-EZH2 axis, but it has not been determine whether WNT-CTNNB1/β-catenin will affect autophagy in these cancer cells [287]. Identification of these kinds of lncRNAs is of importance for their knockdown in further experiments for prostate cancer treatment. For instance, overexpression of the lncRNA *PRRT3-AS1* occurs in prostate cancer cells and tissues. Silencing *PRRT3-AS1* upregulates the expression level of PPARG/PPARγ (peroxisome proliferator activated receptor gamma) that, in turn, inhibits MTOR signaling, leading to autophagy induction and a decrease in viability and survival of prostate cancer cells. In vivo experiments on xenografts in nude mice also confirmed *PRRT3-AS1* silencing and decreased tumor growth [288].

Similar to miRNAs, lncRNAs can target autophagy in affecting the response of prostate cancer cells. The role of the lncRNA *HULC* (hepatocellular carcinoma up-regulated long non-coding RNA) in prostate cancer has been examined in a few studies [289]. Interestingly, currently performed experiments revealed the role of *HULC* as a tumor-promoting factor in prostate cancer. Overexpression of *HULC* enhances prostate cancer metastasis via EMT induction and is correlated with poor prognosis [290]. It has been reported that silencing *HULC* promotes sensitivity of prostate cancer cells to radiotherapy and induces apoptosis and cell cycle arrest (G_0_/G_1_ phase). However, *HULC* overexpression significantly reduces sensitivity to radiotherapy. With regards to the radio-resistance feature of prostate cancer cells, *HULC* induces MTOR signaling, while it diminishes levels of BECN1, leading to autophagy inhibition. Silencing *HULC* triggers autophagy and sensitizes prostate cancer cells to irradiation-mediated apoptosis [291]. Although a few studies have demonstrated autophagy regulation by lncRNAs in prostate cancer, the following points can be concluded:

1. The experiments about lncRNA-mediated autophagy regulation have shown a tumor-suppressor role of autophagy, so that lncRNAs that inhibit autophagy increase prostate cancer progression, and further studies can explore the tumor-promoting role of autophagy and its modulation by lncRNAs;
2. studies have shown that lncRNAs indirectly affect autophagy in prostate cancer via regulating other molecular pathways such as PARP, PI3K-AKT-MTOR, and EZH2. In respect to regulation of miRNAs by lncRNAs via sponging, it can be explored whether lncRNAs can affect miRNAs in targeting autophagy in prostate cancer.

#### CircRNAs

**Fig. 5:**
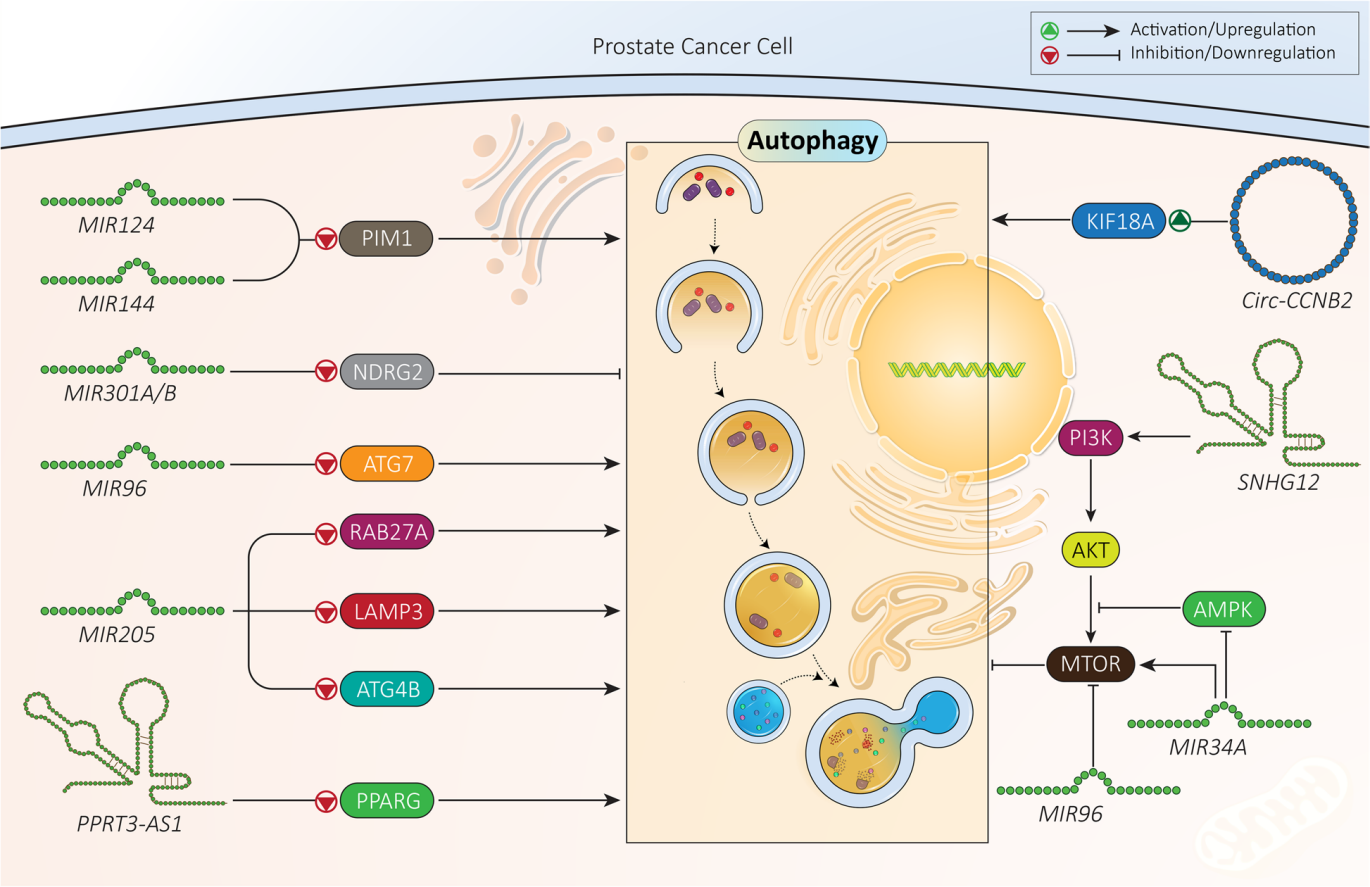
MiRNAs, lncRNAs and circRNAs as regulators of autophagy in prostate cancer. As molecular pathways involved in autophagy regulation by non-coding RNAs have been identified, genetic tools can be utilized for affecting the non-coding RNA-autophagy axis in prostate cancer therapy

Circular RNAs (circRNAs) have a different structure compared to miRNAs and lncRNAs. These ncRNAs form covalently closed loop structures lacking 5′-3′ polarities and polyadenylated tails [292–294]. This special structure of circRNAs makes them more stable than linear RNA molecules, and protects them against degradation by RNA exonucleases [295]. Like miRNAs and lncRNAs, deregulation of circRNAs is observed in prostate cancer. CircRNAs can be considered as potential biomarkers for prostate cancer diagnosis, and they participate in proliferation, migration, and therapeutic response [169, 296, 297]. To date, just one study has evaluated the role of circRNAs in autophagy regulation in prostate cancer, showing that it is still a long journey to reveal the precise role of circRNAs. The circRNA *CCNB2* (cyclin B2) undergoes upregulation in prostate cancer and its silencing promotes sensitivity to irradiation. In inducing the radio-resistance feature of prostate cancer cells, circ-*CCNB2* downregulates the expression level of *MIR30B-5p* via sponging. Then, an increase in the expression level of KIF18A (kinesin family member 18A) occurs to stimulate autophagy, leading to radio-resistance [298]. Overall, fewer studies have been performed regarding the role of circRNAs in prostate cancer compared to miRNAs and lncRNAs. However, based on the capacity of circRNAs to affect miRNA regulation via sponging, and the complicated molecular pathways involved in prostate cancer progression, specific attention should be directed to circRNAs (Fig. 5**,** Table 3).

**Table 3:** Non-coding RNAs regulating autophagy in prostate cancer

Non-coding RNA	Signaling network	Effect on autophagy	Remarks	Refs
*MIR124* *MIR144*	PIM1, autophagy	Inhibition	Hypoxia provides downregulation of *MIR124* and *MIR144* via disrupting DICER1 expression. Enhancing miRNA expression inhibits autophagy via PIM1 downregulation, leading to enhanced radio-sensitivity of prostate cancer cells.	[299]
*MIR301A*/*B*	NDRG2, autophagy	Induction	Increased expression of *MIR301A/B* in the presence of hypoxia. Binding to 3′-UTR of *NDRG2* and reducing its expression. Autophagy induction and enhancing prostate cancer cell viability.	[300]
*MIR96*	MTOR, autophagy ATG7, autophagy	Induction Inhibition	Dual role of *MIR96* in prostate cancer cells and autophagy regulation during hypoxia. Autophagy induction via inhibiting MTOR signaling. Autophagy inhibition via ATG7 downregulation.	[301]
*MIR205*	RAB27A LAMP3	Inhibition	Downregulation of RAB27A and LAMP3 in autophagy inhibition. Impairing proliferation and enhancing chemosensitivity of prostate cancer cells.	[302]
*MIR34A*	AMPK-MTOR-ATG4B	Inhibition	Reducing AMPK phosphorylation. Inducing MTOR signaling. Autophagy inhibition via ATG4B downregulation. Enhancing prostate cancer progression and reducing chemosensitivity.	[303]
LncRNA *SNHG12*	*MIR195*-CCNE1	Inhibition	Overexpression of *SNHG12* in prostate cancer cells. Association with poor prognosis. Reducing *MIR195* expression via sponging. Activation of PI3K-AKT-MTOR signaling. Reducing apoptosis and autophagy.	[304]
LncRNA *PRRT3-AS1*	PPPARG, autophagy	Inhibition	PARP downregulation in impairing autophagy. Enhancing proliferation of prostate cancer cells and reducing apoptosis.	[288]
Circ-*CCNB2*	*MIR30B*-*5p*-KIF18A	Induction	Reducing *MIR30B-5p* expression via sponging. Enhancing KIF18A expression. Autophagy induction and subsequent resistance to radiation therapy.	[298]

#### Other upstream mediators

Previous sections revealed a role for ncRNAs in autophagy regulation in prostate cancer. However, there are other upstream mediators of autophagy in prostate that should be highlighted in directing further research for evaluating other molecular pathways. In this section, our aim is to provide a discussion of some molecular pathways regulating autophagy. KDM4B is a lysine methyltransferase with potential role in AR signaling [305]. KDM4B can induce histone modification to enhance transcriptional activity of AR. Furthermore, KDM4B can increase AR stability via preventing its ubiquitination [306]. Due to the signaling role of AR in prostate cancer progression, KDM4B should have a critical role in this malignant tumor. KDM4B overexpression occurs in prostate cancer cells and is associated with their proliferation. In addition, autophagy activation occurs by KDM4B overexpression in prostate cancer cells. Importantly, KDM4B indirectly induces autophagy via triggering nuclear translation of CTNNB1/β-catenin, leading to prostate cancer progression [307]. CTNNB1/β-catenin is a vital member of WNT signaling and its nuclear translation leads to WNT signaling activation and further induction of other molecular pathways [308]. Although previous experiments revealed a role of CTNNB1/β-catenin in autophagy induction in prostate cancer cells, this pathway can also suppress autophagy in prostate cancer. Nuclear accumulation of CTNNB1/β-catenin caused by nitric oxide (NO) leads to autophagy inhibition in prostate cancer cells [309].

In the section related to prostate cancer metastasis, it was mentioned that SGK1 can mediate autophagy and EMT in prostate cancer cells [149]. SGK1 can also participate in regulating autophagy by affecting the MTOR-FOXO/FOXO3A axis. Briefly, FOXO3 is a Forkhead transcription factor and due to its pro-apoptotic role, is involved in cell cycle arrest and apoptosis [310, 311]. Mechanistically, SGK1 induces MTOR signaling to inhibit FOXO3 phosphorylation, leading to a decrease in apoptosis and autophagy in prostate cancer cells. Silencing SGK1 inhibits MTOR signaling, providing conditions for FOXO3 phosphorylation, and subsequent induction of apoptosis and autophagy in prostate cancer cells [312].

MAPK/JNK is capable of regulating apoptosis after exposure to different stresses [313], and it can also regulate autophagy [314, 315]. In prostate cancer cells, MAPK/JNK signaling induces autophagy as a protective mechanism against celecoxib-mediated apoptosis [316]. This study demonstrates how autophagy and apoptosis interact with each other in prostate cancer cells. As MAPK/JNK-mediated autophagy prevents apoptosis in prostate cancer cells, its inhibition is of importance. Previously, it was shown that KDM4B interaction with AR signaling occurs in prostate cancer. PARK7/DJ-1 is another factor that is vital for AR function and can induce AR signaling [317, 318]. PARK7 prevents the function of the AR inhibitor PIAS2/PIASxa/ARIP3 to induce AR signaling [318]. PARK7 undergoes upregulation in prostate cancer cells and reducing its expression significantly diminishes proliferation and survival. In this way, PARK7 overexpression inhibits MAPK/JNK signaling to prevent BECN1-BCL2 dissociation, resulting in autophagy inhibition and paving the way for prostate cancer progression [319].

Additional experiments have revealed that AR signaling plays a significant role in autophagy induction and prostate cancer progression. SIRT7 (sirtuin 7) is a member of the NAD^+^-dependent deacetylases and its role in cancer is controversial [320, 321]. SIRT7 is responsible for the aggressive behavior of prostate cancer cells, and its downregulation suppresses migration and invasion of cancer cells [322]. SMAD4 is an inhibitor of AR signaling, and its degradation is driven by SIRT7 [323–325]. In prostate cancer cells, SIRT7 undergoes upregulation to inhibit SMAD4, leading to activation of AR signaling, subsequent induction of autophagy and enhancing cancer progression [71]. Overall, different upstream mediators of autophagy such as LC3-II, ATG3, BECN1, EIF4EBP1, SQSTM1 and SIRT1 can be regulated by upstream mediators of autophagy such as TNFAIP8. Based on the role of autophagy in prostate cancer (pro-survival or pro-death), its inhibition or induction can be performed to pave the way into prostate cancer therapy [324, 326].

### Anti-tumor compounds targeting autophagy in prostate cancer

As autophagy plays a significant role in proliferation, metastasis and therapy response of prostate cancer cells, experiments have focused on its targeting this process to affect the progression of cancer cells. In this section, a mechanistic discussion of the role of anti-tumor compounds in autophagy regulation in prostate cancer is provided. Diosgenin is a naturally occurring steroid compound isolated from *Dioscorea nipponica* and has demonstrated high anti-tumor activity in prostate cancer inhibition [327]. Neural precursor cell express NEDD4 (NEDD4 E3 ubiquitin protein ligase), an E3 ligase with a tumor-promoting role in prostate cancer. Diosgenin downregulates NEDD4 expression to stimulate apoptosis and cell cycle arrest [328]. Diosgenin can promote NEDD4 E3 ubiquitin protein ligase Ca^2+^ levels and flux. Furthermore, this compound causes Ca^2+^-independent cell death in prostate cancer cells [329]. Diosgenin administration significantly reduces proliferation and viability of prostate cancer cells via inducing autophagy. In triggering autophagy, diosgenin suppresses PI3K-AKT-MTOR signaling as an upstream mediator of autophagy [330]. In this case, autophagy possesses a tumor-suppressing role. However, when autophagy has a tumor-promoting role, its induction has a negative impact on efficacy of anti-tumor compounds. In prostate cancer suppression, abiraterone downregulates the expression levels of LC3, ATG5 and BECN1 to inhibit autophagy. It is worth mentioning that co-administration of 3-methyladenine as an autophagy inhibitor, enhances the potential of abiraterone in prostate cancer suppression via inducing a further decrease in LC3, ATG5 and BECN1 levels compared to abiraterone alone. This combination remarkably diminishes proliferation and cell viability of prostate cancer cells and stimulates cell cycle arrest at the G_2_/M phase [331].

Eriocalyxin B (EriB), derived from *Isodon eriocalyx* var. *laxiflora*, is a medicinal plant with diverse pharmacological activities in which anti-tumor activity is among them. EriB is a promising candidate in cancer therapy capable of reducing proliferation and cell viability as well as inducing cell cycle arrest and apoptosis via targeting molecular pathways such as STAT3 [332–334]. Through inhibition of the AKT-MTOR axis, EriB stimulates both apoptosis and autophagy in prostate cancer cells. However, with respect to the tumor-promoting role of autophagy in this case, autophagy inhibition by chloroquine potentiates cytotoxicity of EriB against prostate cancer cells [335]. In fact, protective autophagy prevents apoptosis in prostate cancer cells by anti-tumor agents. Along these lines, the autophagy inhibitor bafilomycin A_1_ (BafA1) inhibits autophagy to augment the anti-tumor activity of withaferin A on prostate cancer cells [336].

Due to vital biological roles, it is of great importance to preserve the iron balance in cells [337, 338]. For iron release in cells, iron should first bind to plasma TF (transferrin), and then, iron-bound TF enters cells through endocytosis by binding to TFRC (transferrin receptor) located on the cell surface. When iron levels are low in cells, iron regulatory proteins (IRPs) bind to the iron response elements (IREs) of ferritin and *TFRC* mRNAs. Therefore, TFRC, ferritin and ACO1/iron regulatory protein 1 (aconitase 1) demonstrate the status of the iron level in cells [339]. Increasing evidence demonstrates the role of iron deprivation in apoptosis and autophagy induction in cancer cells [340, 341]. For autophagy induction, curcumin follows a same route. For this purpose, curcumin as a naturally occurring compound derived from *Curcuma longa*, binds to ferric ammonium citrate (FAC). Furthermore, curcumin promotes the expression levels of TFRC and ACO1, demonstrating iron deprivation. However, autophagy induction in these prostate cancer cells enhances their survival and using an autophagy inhibitor potentiates the anti-tumor activity of curcumin [342].

Pathological events in the ER can be evoked courtesy of accumulation of misfolded proteins, oxidative stress induction and impaired calcium homeostasis [343]. Increasing evidence has validated autophagy activation during ER stress to improve ER homeostasis and prevent development of various pathologies. However, when stress is beyond the capacity of autophagy to ameliorate, this condition results in both apoptotic and autophagic cell death [344]. δ-tocoterinol (δ-TT) is a potent anti-tumor agent in prostate cancer suppression. In this way, δ-TT increases the expression level of ER stress markers such as HSPA5/BiP, ElF2A, ERN1 and DDIT3/CHOP. Then, activation of ER stress paves the way for autophagy induction through upregulation of SQSTM1 [345].

One of the important aspects is the close association between levels of autophagy and reactive oxygen species (ROS) [346–349]. Like autophagy, ROS play a dual role in cancer, as tumor-promoting or tumor-suppressing factors [67, 79, 349–352]. Normally, activation of PI3K-AKT signaling upregulates MTOR expression to induce autophagy. Excessive ROS generation inhibits PI3K-AKT signaling to provide MTOR downregulation, resulting in autophagy [353]. Exposing prostate cancer cells to salinomycin induces both apoptosis and autophagy. In triggering apoptosis and autophagy, salinomycin inhibits the PI3K-AKT-MTOR axis, while it induces MAPK14/p38 and MAPK/ERK. Salinomycin also enhances ROS levels. It seems that ROS inhibits autophagy in prostate cancer, because autophagy increases upon the use of an ROS scavenger. More importantly, autophagy inhibition increases ROS production in prostate cancer cells [354].

To promote the potential of anti-tumor agents in prostate cancer treatment, combination cancer therapy is applied. A combination of chloroquine as an autophagy inhibitor and palladium (II) barbiturate complex results in inhibition of PI3K-AKT signaling, MTOR and MAPK14/p38 upregulation to inhibit protective autophagy and potentiate apoptosis induction in prostate cancer cells [355]. Most of the studies demonstrate induction of protective autophagy using anti-tumor compounds [356]. Therefore, if pre-clinical findings are going to be applied in a clinical regimen, using autophagy inhibitors such as chloroquine is recommended.

**Fig. 6:**
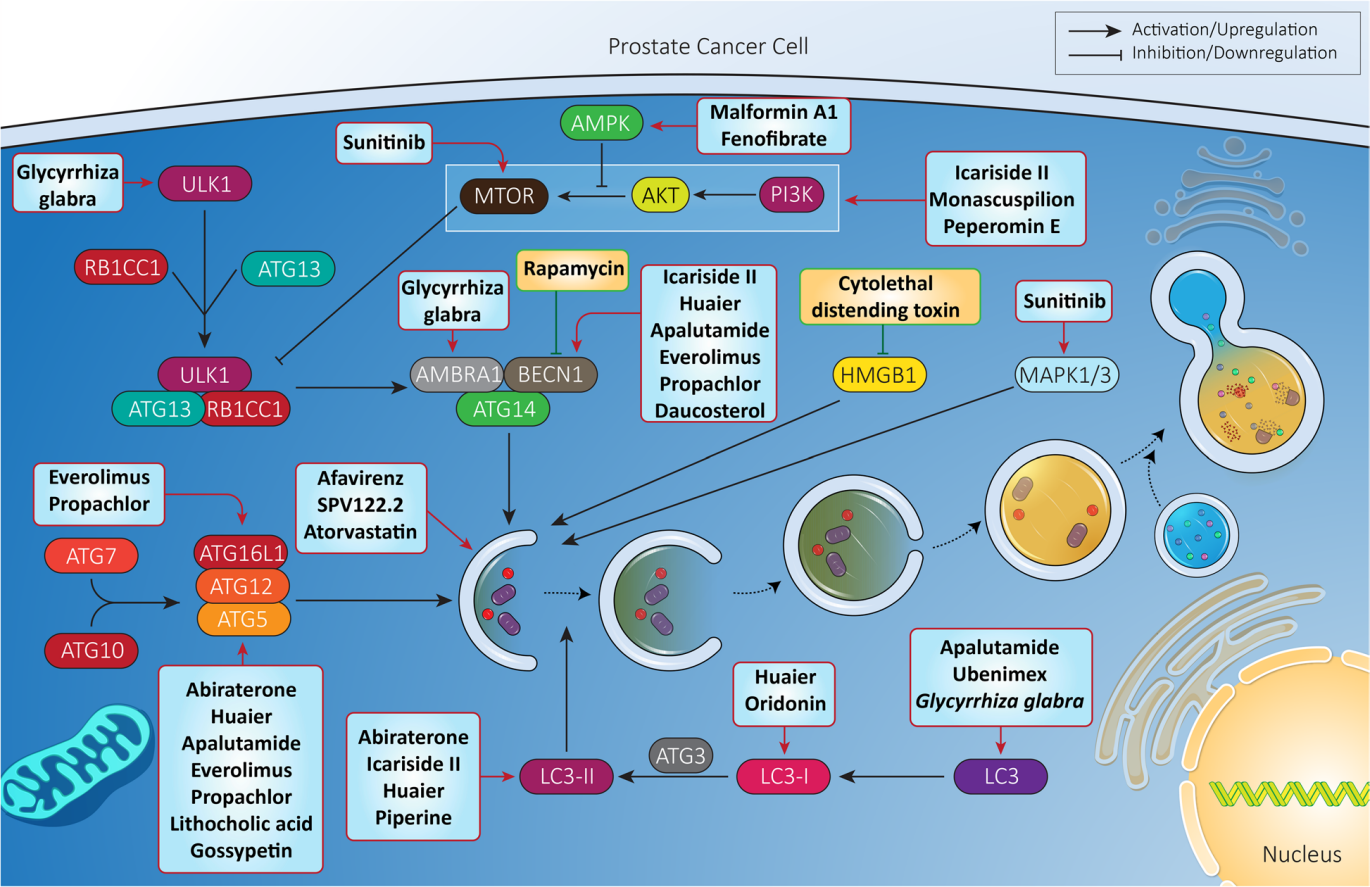
Anti-tumor compounds regulate autophagy in prostate cancer therapy. To provide effective prostate cancer therapy, anti-tumor agents (most being phytochemicals) have been developed for affecting autophagy. Various steps of autophagy and its related molecular pathways are modulated by anti-tumor agents in prostate cancer treatment

In the previous section, it was shown that autophagy is tightly regulated by miRNAs in prostate cancer. Identification of miRNAs and targeting their expression by transfection can pave the way to autophagy regulation and promoting cytotoxicity of anti-tumor compounds against prostate cancer. Celastrol is a bioactive component of *Tripterygium wilfordii* Hook [357] with the capacity of suppressing prostate bone metastasis via decreasing VEGF (vascular endothelial growth factor) secretion [358]. To potentiate the anti-tumor activity of celastrol against prostate cancer cells, nanoparticles such as polymeric and liposomal nanocarriers have been designed [359–361]. New experiments have shown that autophagy and miRNA interaction can determine the response of prostate cancer cells to celastrol therapy. Exposing prostate cancer cells to celastrol stimulates protective autophagy via downregulating *MIR17HG* (miR-17-92a-1 cluster host gene) expression. Further investigation reveals the role of AR as an upstream mediator of *MIR17HG*, so that silencing AR decreases *MIR17HG* expression in prostate cancer cells. Therefore, it seems that celastrol inhibition of AR and its downstream target *MIR17HG* induces protective autophagy [362]. Another experiment reveals the role of AR in regulating *MIR101*. Celastrol administration inhibits the AR-*MIRNA101* axis to induce protective autophagy. Using miRNA replacement therapy and promoting *MIR101* expression inhibits autophagy, potentiating the cytotoxicity of celastrol against prostate cancer cells [363]. Overall, the following points can be concluded (Fig. 6**,** Table 4):

1. Anti-tumor compounds are extensively applied in prostate cancer therapy, and autophagy is one of their targets;
2. most of the administered anti-tumor compounds are phytochemicals;
3. most of the experiments are in agreement with the fact that autophagy induction by anti-tumor compounds functions as a shield against apoptosis;
4. a variety of molecular pathways such as those involving BECN1, ATGs, miRNAs and AR are regulated by anti-tumor compounds in autophagy regulation;
5. using autophagy inhibitors such as chloroquine for autophagy inhibition and enhancing anti-tumor activity in prostate cancer therapy is recommended [391–398].

**Table 4:** Targeting autophagy by anti-cancer compounds in prostate cancer therapy

Anti-tumor compound	Study design	Effect on autophagy	Signaling network	Remarks	Refs
Abiraterone	LNCaP, DU145 and PC3 cells 6, 10 and 16 μM	Induction	ATG5 LC3-II SQSTM1	Enhancing levels of ATG5 and LC3-II. Downregulated SQSTM1. Triggering both autophagy and apoptosis. Inhibition of adaptive autophagy promotes potential of abiraterone in apoptosis induction in prostate cancer cells.	[364]
Icariside II	DU145 cells 0–90 μM	Induction	PI3K-AKT-MTOR	Decreasing proliferation and viability of cancer cells in a time- and dose-dependent manner. Apoptosis and cell cycle arrest induction. Inducing autophagy through PI3K-AKT-MTOR inhibition. Upregulating BECN1 and LC3-II.	[365]
Huaier	PC3 cells 0–8 mg/ml	Induction	ATG3 ATG5 BECN1 LC3-II	Autophagy induction. Decreasing viability and proliferation of cancer cells. Impairing cancer migration. Upregulation of ATG3, ATG5, BECN1 and LC3-II.	[366]
Sunitinib	PC3 and LNCaP cells 5, 10 and 20 μM	Induction	MAPK1/ERK2-MAPK3/ERK1 MTOR	Inhibiting MTOR phosphorylation. Triggering MAPK1-MAPK3 phosphorylation. Autophagy inhibition enhances anti-tumor activity of sunitinib in prostate cancer therapy.	[367]
Apalutamide	LNCaP cells 0–100 μM	Induction	ATG5 BECN1 LC3	Overexpression of ATG5, BECN1 and LC3 to stimulate autophagy. Using autophagy inhibitor enhances efficacy of apalutamide in apoptosis induction in prostate cancer cells.	[365]
NCL1 (histone lysine demethylase 1 inhibitor)	PC3 and 22Rv1 cells PCai1 subcutaneous tumor model 0–100 mM	Induction	–	Triggering both apoptosis and autophagy in prostate cancer cells. Inhibiting autophagy potentiates anti-tumor activity of NCL1, showing anti-tumor activity.	[368]
Reverse transcriptase inhibitors, efavirenz and SPV122.2	PC3 and LNCaP cells 20 μM	Induction	–	Autophagy induction participates in anti-proliferative activity of these agents	[369]
Everolimus Propachlor	PC3 cells 6.15 (propachlor) and 0.70 μM (everolimus)	Induction	BECN1 ATG512–ATG5 complex	Promoting expression level of BECN1. Upregulation of ATG12–ATG5 conjugate. Inducing autophagic cell death. Sensitizing prostate cancer cells to apoptosis.	[370]
Monascuspilion	PC3 cells 0–45 μM	Induction	AKT-MTOR	Suppressing AKT-MTOR axis and subsequent induction of autophagy. Decreasing viability and survival of prostate cancer cells.	[371]
Atorvastatin	PC3 cells and LNCaP cells 5 μM	Induction	BCL2 *MIR182* CDKN1A/p21	BCL2 downregulation, and *MIR182* and CDKN1A upregulation. Exerting anti-proliferation activity. Autophagy induction.	[372]
Lithocholic acid	PC3 and DU-145 cells 5–75 μM	Induction	ATG5	Reducing survival of prostate cancer cells. Autophagy induction via ATG5 upregulation.	[373]
Peperomin E	DU145 cells	Induction	AKT-MTOR	Stimulating both apoptosis and autophagy. Targeting the AKT-MTOR axis. Autophagy plays a protective role. Autophagy inhibition may promote anti-tumor activity of peperomin E against prostate cancer cells.	[374]
Oridonin	PC3 and LNCaP cell lines 0–100 μmol/L	Induction	–	Enhancing conversion of LC3-I to LC3-II. Increasing autophagosome formation. Autophagy induction. CDKN1A upregulation and subsequent apoptosis in prostate cancer cells. Autophagy inhibition reverses CDKN1A upregulation.	[375]
Phenethyl isothiocyanate	PC-3 and LNCaP cells 5 μM	Induction	–	The significant decrease occurs in progression of prostate cancer cells exposed to PEITC The increased levels and generation of ROS by PEITC are responsible for triggering autophagy in prostate cancer cells	[376]
Lu01-M (a secondary metabolite)	PC3, DU145, and LNCaP cells 1.56, 3.125 and 6.25 μg/mL	Induction	–	The Lu01-M triggered DNA damage, apoptosis, necrosis and autophagy in prostate cancer cells, but autophagy function was pro-survival, and its inhibition should be considered in next studies	[374]
Plectranthoic acid	DU145, CW22Rν1, PC3, NB26, and A375 cells 20–40 μM	Induction	MTOR	Plectranthoic acid inhibits phosphorylation of mTOR signaling to induce autophagy and along with apoptosis are responsible for reducing survival of prostate cancer cells	[377]
Curcumin	22rv1, LNCaP, DU145 and PC-3 cells 10, 20, 50, 75, 100 μM	Induction	WNT	Inhibiting Wnt signaling and preventing interaction of β-catenin and TCF-4 protein in triggering autophagy and reducing prostate cancer progression	[378]
Zoledronic acid	PC-3, DU-145, LNCaP and CRW22Rv1 cell lines 100 μM	Induction	–	Inducing autophagy and apoptosis decrease prostate cancer progression, and using autophagy and apoptosis inhibits promotes proliferation and viability of tumor cells	[379]
Cysmethynil	PC-3 cells 0–50 μM	Induction	MTOR	Mediating cell death and G1 phase arrest Triggering autophagic cell death	[380]
Marchantin M	PC-3 cells 2.5, 5, 10 and 20 μM	Induction	PI3K-AKT-MTOR EIF2AK3/PERK-EIF2A	Triggering autophagic cell death in prostate tumor cells via suppressing PI3K-AKT-MTOR axis and inducing PRK/elF2α axis	[381]
*Glycyrrhiza glabra*	PC3 cells 0–100 nM	Induction	LC3A ULK1 AMBRA1	Triggering autophagy via enhancing expression levels of LC3A, ULK1 and AMBRA1. Anti-tumor activity of autophagy. Apoptosis induction.	[382]
Fenofibrate	DU145 and PC3 cells	Induction	AMPK	AMPK phosphorylation and subsequent induction of autophagy. Increased sensitivity of prostate cancer cells to docetaxel chemotherapy.	[383]
Gossypetin	PCa, LNCaP and DU145 cell lines 0–100 μM	Induction	ATG5	ATG5 upregulation and subsequent triggering of autophagy. Reducing tumor growth in vivo.	[384]
Docetaxel	PC3 and LNCaP cell lines 1, 10 and 100 nM	Inhibition	PI3K-AKT-MTOR	Inhibiting PI3K expression. Autophagy suppression. Enhancing apoptosis in cancer cells.	[385]
Hydroxytyrosol	PC3 cells	Inhibition	–	Increasing ROS levels in autophagy impairment to diminish proliferation and viability of prostate cancer cells.	[386]
Cytolethal distending toxin	LAPC4 PCa cells 0–500 nM	Inhibition	MYC HMGB1	Preventing radiation-mediated autophagy via downregulating MYC expression. Further inhibition of autophagy by HMGB1 inhibition.	[387]
Rapamycin	PC3, DU145 and LNCaP cells	Inhibition	BECN1	Enhancing potential of radiotherapy in prostate cancer suppression. Decreased expression level of BECN1. Providing radio-sensitivity via autophagy inhibition.	[388]
Propranolol	H33258 cells 100 μM	Inhibition	–	Suppressing prostate cancer progression. Enhancing autophagosome accumulation due to autophagy blockade. Promoting potential of 2-deoxyglucose in glycolysis inhibition and preventing prostate cancer progression. Autophagy inhibition exacerbates ER stress.	[389]
Pyroglutamate-modified peptide (pE-K092D)	MDA-Pca-2b cell line	Inhibition	–	Proliferation inhibition, cytoskeleton disruption and autophagy inhibition are responsible for decreased progression of prostate cancer cells	[390]

### Gene therapy

In earlier sections, it was discussed that anti-tumor compounds are efficient in autophagy regulation and suppressing prostate cancer progression. Now, the question that comes into mind is whether there any genetic tools with efficiency in autophagy regulation. As experiments have identified upstream mediators of autophagy in prostate cancer, and due to the presence of genetic tools such as siRNA, shRNA and the CRISPR-Cas9 system, molecular pathways can be targeted in autophagy regulation that affect the progression of prostate cancer cells. To date, siRNA has been extensively applied in autophagy regulation and cancer therapy. For instance, co-application of *ATG7* siRNA and docetaxel is beneficial in breast cancer therapy [399]. SiRNA is the most well-known genetic tool utilized for affecting autophagy in prostate cancer. The siRNA application helps in inhibiting or inducing autophagy and determining its exact role—as a tumor promoter or tumor suppressor [223]. Furthermore, siRNA has helped reveal the apoptosis and autophagy interaction in prostate cancer. Previously, it was shown that tumor-suppressor autophagy can enhance the sensitivity of prostate cancer cells to apoptosis. Thus, does autophagy precede apoptosis in prostate cancer or vice versa? Overexpression of BIRC5/survivin prevents apoptosis in prostate cancer cells and is a positive factor in their progression and survival. BIRC5/survivin downregulation using siRNA sensitizes prostate cancer cells to apoptosis and autophagy. Suppressing early or later events of autophagy alleviates apoptosis induction in prostate cancer cells, showing that for apoptosis, the first step is autophagy induction. In fact, autophagy precedes apoptosis [400]. Noteworthy, siRNA has also been advantageous in elucidating the ER stress and autophagy interaction in prostate cancer. Exposure to cadmium is associated with prostate cancer tumorigenesis. Cadmium increases ROS generation to induce ER stress, leading to autophagy impairment and paving the way for prostate cancer development. ATF4 downregulation by siRNA inhibits ER stress and autophagy impairment, showing that ER stress stimulation is vital for triggering defects in autophagy [401]. Overall, experiments agree with the fact that upstream mediators of autophagy can be suppressed using siRNA, and this tool is also beneficial in revealing the relationship of autophagy with other molecular pathways and mechanisms in prostate cancer [402–406]. ShRNA is another tool that can be utilized for autophagy induction and suppressing prostate cancer progression. The function of shRNA is similar to siRNA, so that after entering cells, shRNA generates hairpin RNA that translocates to the cytoplasm, undergoing cleavage by DICER enzyme, producing siRNA and subsequent incorporation in the RNA-induced silencing complex (RISC) for gene silencing [407]. Overexpression of IGF1R (insulin like growth factor 1 receptor) promotes prostate cancer growth and progression. For exerting its carcinogenesis impact, IGF1R inhibits autophagy. IGF1R inhibition using shRNA results in autophagy induction through promoting expression levels of LC3B, leading to decreased prostate cancer progression [408]. More experiments are required to demonstrate the potential of other genetic tools such as the CRIPSR-Cas9 system in autophagy regulation and suppressing prostate cancer progression.

### Nanotherapeutics and biological vectors

Effective treatment of cancer depends on combining different disciplines to augment the capacity of current therapeutic strategies. In the previous section, it was shown that autophagy plays a significant role in different aspects of prostate cancer cells such as proliferation, metastasis, and therapy response. To modulate autophagy, anti-tumor compounds have been utilized with promising results in prostate cancer treatment. Furthermore, gene therapy has been applied in regulating autophagy and prostate cancer progression. However, each of the strategies suffer from some drawbacks. Most of the anti-tumor compounds applied in prostate cancer treatment via autophagy regulation, are phytochemicals and poor bioavailability is the major issue related to plant derived-natural compounds, limiting their therapeutic impacts [409]. SiRNA application is restricted in autophagy regulation and prostate cancer treatment due to its degradation and off-target effects [410, 411]. Therefore, there is a need for development of novel strategies to improve the efficacy of biology-based methods. In this case, bioengineering comes into view and a wide variety of experiments have applied nanotechnological strategies for suppressing proliferation and metastasis of prostate cancer cells, gene, and drug delivery, and finally imaging [412–417]. Nanoparticles can regulate autophagy in prostate cancer via affecting molecular pathways and lysosomes. It has been shown that exposing prostate cancer cells to silver nanocarriers impairs the integrity of the lysosomal membrane, diminishes the number of lysosomes, and prevents lysosomal protease activity, leading to autophagy flux inhibition. Furthermore, silver nanoparticles induce the AMPK-MTOR axis in suppressing autophagy in prostate cancer cells [418]. In fact, attention should be directed towards regulating autophagy by nanoparticles, while these carriers are applied for gene delivery in prostate cancer therapy [419]. Regardless of nanoparticle-mediated regulation of autophagy in prostate cancer, nanocarriers can provide a platform for gene delivery in prostate cancer treatment. In one experiment, chitosan nanoparticles have been prepared for *MIR34A* delivery to affect autophagy in prostate cancer treatment. *MIR34A* is a tumor-suppressor factor in prostate cancer and mediating its delivery by chitosan nanoparticles promotes its potential in cancer suppression. Upon *MIR34A* delivery, a significant increase occurs in apoptosis induction in prostate cancer cells and their viability and survival decrease. Further investigation revealed the relationship between autophagy and *MIR34A* cell death in prostate cancer cells. *MIR34A*-loaded chitosan nanoparticles stimulate autophagy in prostate cancer cells independent of BECN1, ATG4, ATG5 and ATG7, known as non-canonical autophagy. Autophagy induction by *MIR34A* negatively affects survival of prostate cancer cells, so that apoptosis inhibition does not block the anti-proliferative activity of *MIR34A*-mediatd autophagy [420]. Inorganic carbon nanomaterials have shown anti-tumor activity against prostate cancer cells via autophagy and apoptosis induction [421]. To date, a few studies have investigated nanoparticles and autophagy regulation in prostate cancer, but as autophagy is a vital process in prostate cancer, further experiments can focus how delivery of anti-tumor compounds by nanocarriers or their co-delivery with genetic tools can affect autophagy in favor of prostate cancer treatment.

**Fig. 7:**
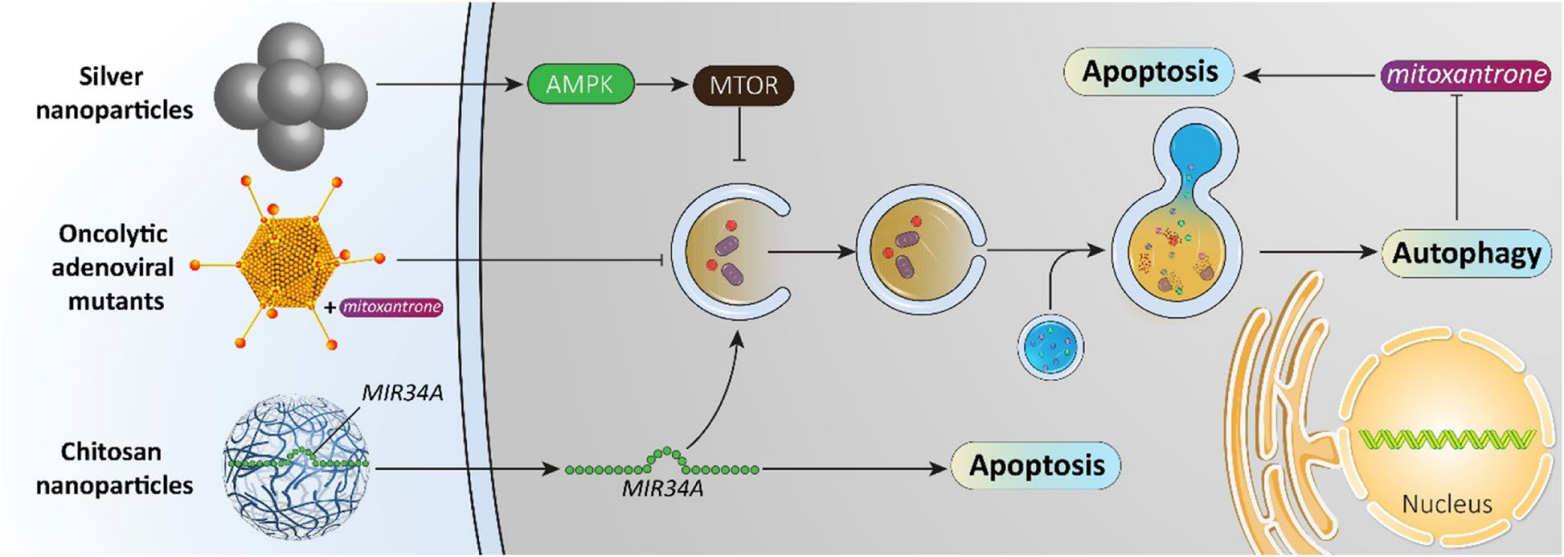
The nanotherapeutics and biological vectors in regulating autophagy for prostate cancer therapy

Oncolytic adenoviral mutants are ideal candidates in the treatment of solid tumors, and their combination with irradiation or anti-tumor compounds leads to remarkable reduction in cancer progression [422, 423]. Due to high potency and selectivity, adenoviral mutants with deletions in the viral E1ACR2-region are extensively applied in cancer therapy in clinical treatment [424–427]. In some prostate cancer therapy, mitoxantrone is applied, but autophagy induction reduces its potential in cancer treatment. Application of oncolytic mutant Ad∆∆ (E1B19K- and E1ACR2-deleted) inhibits autophagy to promote the potential of mitoxantrone in prostate cancer apoptosis. Further inhibition of ATG7 also enhances the efficacy of mitoxantrone in prostate cancer apoptosis, demonstrating a protective role of autophagy in prostate cancer and its inhibition by this oncolytic virus (Fig. 7) [428].

### Other kinds of autophagy

#### Mitophagy

Mitophagy is a special type of autophagy responsible for degradation of damaged mitochondria [429]. The mitochondrial quality is determined via its contents [430]. Recently, studies have focused on mitophagy in disorders such as neurodegenerative disorders in which some mitophagy proteins including PINK1 (PTEN induced kinase 1) or PRKN/Parkin undergo mutation [431–433]. Mitophagy plays a significant role in cancer [434]. Autophagy inhibition results in an increase in bone metastasis of breast cancer cells [435]. Noteworthy, the role of mitophagy in prostate cancer has been examined. At the first step, it seems that mitophagy induction is advantageous for sensitizing prostate cancer cells to apoptosis. Exposing prostate cancer cells to abiraterone and MDC3100 results in downregulation of mitochondrial proteins such as FXN (frataxin), ACO2 and TOMM20, mitochondrial swelling, mitochondrial depolarization and decreased mitochondrial DNA copy number. These effects result in mitophagy that is associated with apoptosis and reduced proliferation of prostate cancer cells [436]. Metabolic vulnerability of prostate cancer cells is a negative factor for their survival. CAV1 (caveolin 1) alters lipid metabolism in prostate cancer cells that, subsequently, stimulates mitophagy as a lethal process [437]. Although much emphasis was placed on the anti-tumor role of mitophagy, additional investigation reveals a tumor-promoting role of mitophagy in prostate cancer that should be highlighted in directing further experiments. It has been shown that autophagy and mitophagy induction by the integrin ITGA6/a6b1 and the receptor BNIP3 significantly enhances survival and viability of prostate cancer cells [438]. Therefore, more experiments are required to reveal the role of mitophagy in prostate cancer. Agents capable of impairing mitochondrial function can promote mitophagy induction. Retigeric acid B as a potent anti-tumor agent, enhances ROS generation in prostate cancer cells to impair the normal function of mitochondria, resulting in mitophagy and autophagy. Autophagy inhibition increases apoptosis in prostate cancer cells [439]. Although a few studies have examined the role of mitophagy in prostate cancer, more efforts are warranted to decipher how mitophagy participates in the therapy response of prostate cancer cells, and how this important mechanism can affect senescence induction.

#### Lipophagy

**Fig. 8:**
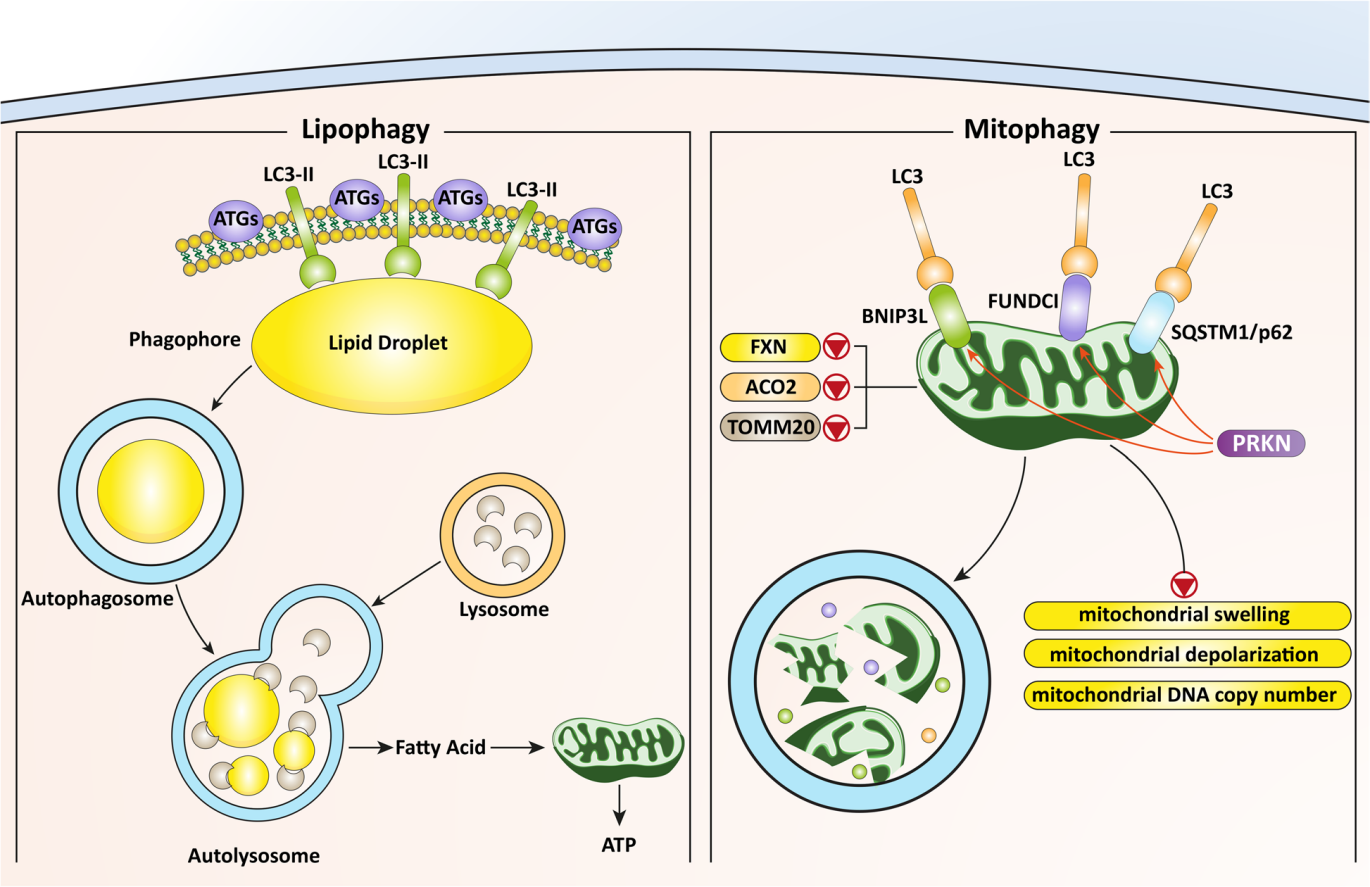
Other types of autophagy in prostate cancer

Lipophagy is a form of autophagy first characterized in 2009. Compared with other types of autophagy, lipophagy has not been well elucidated, and the terminology primarily depicts the degradation of lipid droplets (LDs) by autophagy [440]. Lipophagy is like non-specific canonical autophagy in terms of macro- and micro-based mechanisms. Macrolipophagy involves delivery of LDs to lysosomes through autophagosomes and subsequent degradation. However, in microautophagy, lysosomes directly and transiently interact with LDs. Noteworthy, CMA is not directly involved in lipohagy [441]. To date, only two studies have examined role of lipophagy in prostate cancer. Typically, cancer cells undergo senescence upon therapy. Exposure of prostate cancer cells to *Abrus* agglutinin (AGG) leads to lipophagy-induced accumulation of fatty acids, and a reduction in the number of LDs. Using a lysosomal acid lipase inhibitor prevents lipophagy-mediated senescence, showing that lipophagy is vital for this process. Mechanistically, AGG stimulates cytoplasmic SIRT1 that in turn, deacetylases LAMP1 (lysosomal associated membrane protein 1) on lysine residues of the cytosolic domain, resulting in lipophagy-mediated senescence in prostate cancer cells [442]. Another study revealed that autophagy can contribute to LDs depletion in prostate cancer cells and promote prostate cancer growth. Autophagy inhibition using pharmacological or genetic interventions lead to a decrease in LDs depletion and cell growth. Although this study does not specifically examine lipophagy, it involved autophagy in the degradation of LDs and thus probably should be considered as lipophagy (Fig. 8) [443].

### Role of autophagy in prognosis and diagnosis

Importantly, autophagy can be considered as a reliable biomarker for prostate cancer diagnosis and prognosis. The aggressive behavior of prostate cancer cells has forced studies to focus on detecting biomarkers [444, 445]. Such biomarkers can provide a diagnosis map for prostate cancer and can help practitioners in providing a prognosis. In this section, our aim is to show how autophagy can be utilized as a diagnostic and prognostic tool in prostate cancer patients. A recent experiment has evaluated 495 prostate cancer tissues in terms of expression of differentially expressed autophagy-related genes (DEARGs). These genes are responsible for autophagy regulation and subsequent impact on resistance of prostate cancer to platinum-containing compounds and EGFR tyrosine kinase inhibitors. Among them, five autophagy-related genes (ARGs) including *ATG9B*, *DNAJB1*, *HSPB8*, *NKX2-3* and *TP63* demonstrate remarkable association with prostate cancer development [446]. Further investigation demonstrates that autophagy can also be considered as a prognostic factor in prostate cancer. For this purpose, a recently conducted experiment has shown that overexpression of ARGs such as FAM215A, MYC and FADD can provide a poor prognosis and low overall survival rate of prostate cancer patients [447]. An interesting study performed in hospitals in Guangzhou, China revealed the reliability of MAP1S as biomarker in prostate cancer. MAP1S provides a connection between autophagic components with microtubules and mitochondria, en route to induction of autophagy. Further examination noted prostate cancer patients with high level of MAP1S demonstrate better prognosis compared to those with low level of MAP1S [448].

**Fig. 9:**
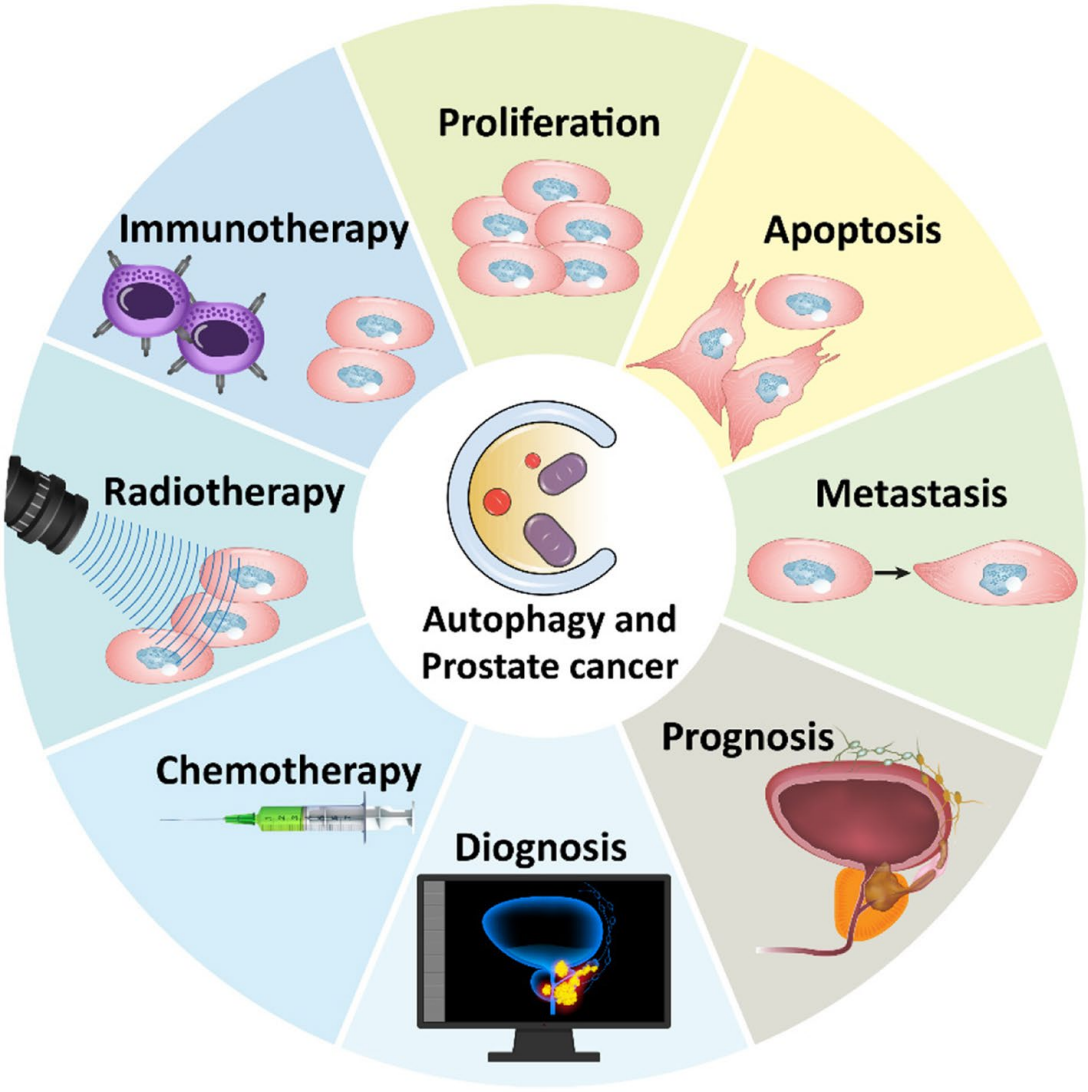
The autophagy mechanism signature in prostate cancer

LRPPRC (leucine rich pentatricopeptide repeat containing) is considered as a negative regulator of autophagy and demonstrates upregulation in prostate cancer tissues. An analysis has shown that LRPPRC is overexpressed in 75% of prostate cancer patients, while it has low expression in 10% of prostate cancer patients. As an autophagy inhibitor, upregulation of LRPPRC reveals poor prognosis, low overall survival, and resistance to hormone therapy of prostate cancer [449]. Another experiment on 458 prostate cancer patients demonstrates association of ATG16L1 polymorphism (rs78835907) with recurrence and poor survival. In fact, ATG16L1 as an autophagy regulator, undergoes downregulation in patients with poor survival [450]. Previously, it was discussed that miRNAs can regulate autophagy in prostate cancer. A clinical study has confirmed miRNA and ARGs interaction in prostate cancer and their biomarker role. One of the strategies for prostate cancer immunotherapy is using PDCD1/PD-1 (programmed cell death 1) inhibitors. Briefly, PDCD1 participates in providing immune evasion and reducing anti-tumor immunity [451]. An ARG, known as NKX2-3 demonstrates overexpression in prostate cancer patients, and is associated with poor overall survival, lymph node metastasis and reduced capacity of PDCD1 inhibitors. Further studies reveal an interaction between *MIR205* and NKX2-3 that determines the therapy response of prostate cancer patients [452]. Therefore, autophagy and its related genes are potential biomarkers for prostate cancer diagnosis and prognosis [446, 453, 454]. Figure 9 provides a schematic representation of autophagy mechanism involvement in various aspects of prostate cancer from proliferation and metastasis to therapy resistance and prognostic signature.

### Paving the way for clinical translation

Our aim in this review was to recap how autophagy participates in proliferation, metastasis, and therapy response of prostate cancer cells. Emphasis was put on molecular signaling pathways regulating autophagy and how they may be altered by anti-tumor compounds, gene therapy, and bioengineering strategies. To improve our understanding towards the role of autophagy in prostate cancer, a section examining autophagy as a diagnostic and prognostic tool was included. Noteworthy, previous steps for introducing autophagy into clinical treatment have been made, and, currently, autophagy is considered as a reliable biomarker for prostate cancer diagnosis. However, there is no clinical trial related to targeting autophagy for treatment of prostate cancer patients. For instance, chemotherapy failure is a common phenomenon in prostate cancer patients, owing to a significant role of autophagy in drug resistance and radio-resistance. Therefore, novel therapeutics can be developed in this case. In addition to anti-tumor compounds targeting autophagy, genetic tools such as siRNA, shRNA and CRISPR-Cas9 can be applied in this case. Further improvement can be made via introducing bioengineering for improving the capacity in autophagy regulation for treatment of prostate cancer patients. However, we are still at a beginning point and further studies will shed more light on targeting autophagy in prostate cancer therapy.

## Conclusion and remarks

Effective management of prostate cancer requires a better understanding of molecular mechanisms involved in the onset and progression of prostate cancer. Among various mechanisms, autophagy possesses a dual role in cancer, and can either promote or suppress cancer progression. This review aimed to evaluate the role of autophagy in prostate cancer, as the most malignant tumor in men. Proliferation and metastasis of prostate cancer cells are tightly regulated by autophagy. Through manipulation of autophagy (downregulation or upregulation based on its role), proliferation and metastasis can be regulated for prostate cancer cells. Metabolic reprogramming is extensively adopted by prostate cancer cells in enhancing their progression. It seems that autophagy inhibition (for instance, by DNM1L upregulation), accelerates metabolic reprogramming in favor of prostate cancer progression. In this case, stimulating autophagy is of importance. Since autophagy determines growth and migration of prostate cancer cells, this molecular mechanism can be related to therapy response of prostate cancer cells. Many experiments have demonstrated the role of autophagy in chemotherapy and radiotherapy, as well as its modulation as an appropriate tool. During the initiation of prostate cancer, the autophagy machinery function as a pro-apoptotic; however, once it progresses, autophagy act as a proliferator, more specifically in CRPC and androgen ablation therapies.

Among the various molecular pathways regulating autophagy in prostate cancer, ncRNAs including miRNAs, lncRNAs and circRNAs have been investigated to a greater extent compared to other molecular pathways. It is worth mentioning that like autophagy, a ncRNA such as miRNA or lncRNA can act as a double-edged sword in cancer. This feature significantly complicates autophagy regulation in prostate cancer, and further efforts in developing novel therapeutics. According to current review, autophagy function in prostate cancer is context-dependent and it may action as pro-survival or pro-death mechanism in each stage of prostate cancer. Therefore, extensive research in future can reveal and highlight this action of autophagy.

The important hint is that experiments have focused on using anti-tumor compounds for autophagy regulation in prostate cancer treatment, and, also, using genetic tools such as siRNA and shRNA. However, these strategies are not completely effective in prostate cancer treatment, unless their efficiency is improved using nanoparticles. There are currently no experimental data about gene delivery by nanoparticles for autophagy regulation in prostate cancer treatment and future studies can focus on this aspect. Furthermore, autophagy has been applied as a biomarker for diagnosis and prognosis of prostate cancer. This is a milestone in progress in introducing autophagy into the clinic. Finally, for improving autophagy targeting in prostate cancer therapy in clinical treatment, the recommendations in section 3.10 should be considered.

There are some limitations related to current works that should be considered in future experiments. Most of the experiments have focused on phytochemicals, while their clinical application is restricted. A few small molecules have been examined for targeting autophagy in prostate cancer therapy. Hence, future studies should focus on developing new small molecular targeting autophagy regulators such as BECN1 and ATGs, among others. Although autophagy function in chemotherapy and radiotherapy responses of prostate cancer cells has been investigated, more anti-cancer agents should be tested. Furthermore, association between autophagy and apoptosis in chemoresistance/chemosensitivity should be highlighted, as apoptosis is the most well-known mechanism affected by anti-cancer agents. Another limitation is related to therapeutic targeting of genes for autophagy regulation in prostate cancer treatment. Based on the discussions, ncRNAs and various kinds of molecular pathways have capacity of autophagy regulation. However, genetic tools such as CRISPR-Cas9 system, siRNA and shRNA have not been fully employed in autophagy regulation in prostate cancer suppression. Therefore, at the first step, future experiments should focus on targeting specific genes regulating autophagy such as BECN1 and ATGs. Despite efforts for using nanoparticles and biological vectors in autophagy regulation and suppressing prostate cancer progression, there are still some limitations. The studies have only focused on carbon-based and metal-based nanomaterials for autophagy regulation in prostate cancer treatment. Other kinds of nanostructures such as lipid-based nanoparticles and polymeric nanostructures should also be employed. Furthermore, nanostructures can be utilized for improving potential of genetic tools in autophagy regulation for prostate cancer suppression.

## Data Availability

Not applicable.

## Abbreviations

CRPC: Castration-resistance prostate cancer
AR: Androgen receptor
REST: RE1 silencing transcription factor
CMA: Chaperone-mediated autophagy
ATG: Autophagy related
PI3K: Phosphoinositide 3-kinase
ER: Endoplasmic reticulum
MAP 1LC3/LC3: Microtubule associated protein 1 light chain 3
MTOR: Mechanistic target of rapamycin kinase
AMPK: AMP-activated protein kinase
LAMP: Lysosomal associated membrane protein
PCD: Programmed cell death
MHCI: Major histocompatibility complex class I
GABARAPL1: GABA type A receptor associated protein like 1
HIF1A/HIF-1α: Hypoxia inducible factor 1 subunit alpha
FGFs: Fibroblast growth factors
DNM1L/DRP1: Dynamin 1 like
HSD17B4: Hydroxysteroid 17-beta dehydrogenase 4
MAPK/JNK: Mitogen-activated protein kinase
ECM: Extracellular matrix
EMT: Epithelial-to-mesenchymal transition
WFDC2: WAP four-disulfide core domain 2
EGFR: Epidermal growth factor receptor
TME: Tumor microenvironment
ADT: Androgen-deprivation therapy
HDAC6: Histone deacetylase 6
TGFB/TGF-β: Transforming growth factor beta
NFKB/NF-κB: Nuclear factor kappa B
STAT3: Signal transducer and activator of transcription 3
miRNAs: MicroRNAs
SGK1: Serum/glucocorticoid regulated kinase 1
siRNA: Small interfering RNA
shRNA: Short hairpin RNA
WWOX: WW domain containing oxidoreductase
NPRL2: NPR2 like, GATOR1 complex subunit
MSCs: Mesenchymal stem cells
HDAC3: Histone deacetylase 3
CP: Cisplatin
AMBRA1: Autophagy and beclin 1 regulator 1
PARP: Poly(ADP-ribose) polymerase
ASAH1/acid ceramidase: N-acylsphingosine amidohydrolase 1
HMGB1: High mobility group box 1
TPD52/PrLZ/prostate leucine zipper: tumor protein D52
STK11/LKB1: Serine/threonine kinase 11
PTAFR: Platelet activating factor receptor
GPCR: G-protein-coupled receptor
ncRNAs: Non-coding RNAs
mRNA: Messenger RNA
3’-UTR: 3’-untranslated region
PTEN: Phosphatase and tensin homolog
RELN: Reelin
PHLPP2: PH domain and leucine rich repeat protein phosphatase 2
TP53INP1: Tumor protein p53 inducible nuclear protein 1
SP1: Sp1 transcription factor
PKM2: Pyruvate kinase M1/2; SIRT1 sirtuin 1
ERN1/IRE1: Endoplasmic reticulum to nucleus signaling 1
*SNHG1*: Small nucleolar RNA host gene 1
EZH2: Enhancer of zeste polycomb repressive complex 2 subunit
PPARG/PPARγ: Peroxisome proliferator activated receptor gamma
*HULC*: Hepatocellular carcinoma up-regulated long non-coding RNA
circRNAs: Circular RNAs
circ-*CCNB2*: CircRNA cyclin B2
KIF18A: Kinesin family member 18A
NO: Nitric oxide
MAPK: Mitogen-activated protein kinase
SIRT7: Sirtuin 7
NEDD4: NEDD4 E3 ubiquitin protein ligase
EriB: Eriocalyxin B
BafA1: Bafilomycin A_1_
TF: Transferrin
TFRC/TfR1: Transferrin receptor
IRPs: Iron regulatory proteins
IREs: Iron response elements
ACO1/IRP1: Aconitase 1
FACs: Ferric ammonium citrate
αTT: α-tocotericol
ROS: Reactive oxygen species
VEGF: Vascular endothelial growth factor
RISC: RNA-induced silencing complex
IGF1R: Insulin like growth factor 1 receptor
PINK1: PTEN induced kinase 1
CAV1: Caveolin 1
LDs: Lipid droplets
AGG: *Abrus* agglutinin
DEARGs: Differentially expressed autophagy-related genes
ARGs: Autophagy-related genes
LRPPRC: Leucine rich pentatricopeptide repeat containing
PDCD1/PD-1: Programmed cell death 1
